# Cometary Comae-Surface Links: The Physics of Gas and Dust from the Surface to a Spacecraft

**DOI:** 10.1007/s11214-020-00744-0

**Published:** 2020-11-06

**Authors:** Raphael Marschall, Yuri Skorov, Vladimir Zakharov, Ladislav Rezac, Selina-Barbara Gerig, Chariton Christou, S. Kokou Dadzie, Alessandra Migliorini, Giovanna Rinaldi, Jessica Agarwal, Jean-Baptiste Vincent, David Kappel

**Affiliations:** 1 Southwest Research Institute, 1050 Walnut St, Suite 300, Boulder, CO 80302 USA; 2 International Space Science Institute (ISSI), Hallerstrasse 6, 3012 Bern, Switzerland; 3 Institut für Geophysik und extraterrestrische Physik, Technische Universität Braunschweig, Mendelssohnstr. 3, 38106 Braunschweig, Germany; 4 Max-Planck-Institut für Sonnensystemforschung, Justus-von-Liebig-Weg 3, 37077 Göttingen, Germany; 5 IAPS-INAF, via Fosso del Cavaliere, 100, 00133 Rome, Italy; 6 Physikalisches Institut, University of Bern, Sidlerstr. 5, 3012 Bern, Switzerland; 7 NCCR PlanetS, Sidlerstrasse 5, 3012 Bern, Switzerland; 8 School of Engineering and Physical Sciences, Heriot-Watt University, Edinburgh, EH14 4AS Scotland UK; 9 Deutsches Zentrum für Luft- und Raumfahrt (DLR), Institut für Planetenforschung, Rutherfordstrasse 2, 12489 Berlin, Germany; 10 Institute of Physics and Astronomy, University of Potsdam, Potsdam-Golm, Germany

**Keywords:** Comets, Coma, Gas, Dust, Dynamics, Modelling, Inversion

## Abstract

A comet is a highly dynamic object, undergoing a permanent state of change. These changes have to be carefully classified and considered according to their intrinsic temporal and spatial scales. The Rosetta mission has, through its contiguous in-situ and remote sensing coverage of comet 67P/Churyumov-Gerasimenko (hereafter 67P) over the time span of August 2014 to September 2016, monitored the emergence, culmination, and winding down of the gas and dust comae. This provided an unprecedented data set and has spurred a large effort to connect in-situ and remote sensing measurements to the surface. In this review, we address our current understanding of cometary activity and the challenges involved when linking comae data to the surface. We give the current state of research by describing what we know about the physical processes involved from the surface to a few tens of kilometres above it with respect to the gas and dust emission from cometary nuclei. Further, we describe how complex multidimensional cometary gas and dust models have developed from the Halley encounter of 1986 to today. This includes the study of inhomogeneous outgassing and determination of the gas and dust production rates. Additionally, the different approaches used and results obtained to link coma data to the surface will be discussed. We discuss forward and inversion models and we describe the limitations of the respective approaches. The current literature suggests that there does not seem to be a single uniform process behind cometary activity. Rather, activity seems to be the consequence of a variety of erosion processes, including the sublimation of both water ice and more volatile material, but possibly also more exotic processes such as fracture and cliff erosion under thermal and mechanical stress, sub-surface heat storage, and a complex interplay of these processes. Seasons and the nucleus shape are key factors for the distribution and temporal evolution of activity and imply that the heliocentric evolution of activity can be highly individual for every comet, and generalisations can be misleading.

## Introduction and Historical Context

The Rosetta mission has, through its contiguous in-situ and remote sensing coverage of comet 67P/Churyumov-Gerasimenko (hereafter 67P) over the time span of August 2014 to September 2016, monitored the emergence, culmination, and winding down of the gas and dust comae. This provided an unprecedented data set. One of five prime goals the mission addressed from the original Rosetta announcement of opportunity (RO-EST-AO-0001, 1st March 1995; see also Keller and Kührt (2020)) was to study “The development of cometary activity and the processes in the surface layer of the nucleus and in the inner coma”. The processes involved in the gas and dust release and subsequent expansion have thus been intensely studied. Linking the observations of the comae to the diverse morphology of the nucleus as described in this book in El-Maarry et al. (2019) and the local manifestations of activity as described in this book in Vincent et al. (2019) is therefore of particular interest. Even though understanding this link of the coma to the surface is of vital importance to understanding cometary activity on a more basic level this has turned out to be immensely challenging, even with a large data set as provided by the in-situ and remote sensing instruments. There are fundamental reasons for this. First, remote sensing instruments (e.g. visible to infrared imaging systems and spectrometers) all provide line-of-sight (LOS) measurements of the comae which are thus naturally a convolution of the gas or dust distribution around the nucleus and the viewing geometry. In addition, long integration times are often necessary for a sufficiently high signal-to-noise ratio (S/N). This complicates the interpretation if the integration time exceeds a significant fraction of e.g. the rotation period of the nucleus, therefore additionally convolving with the temporal evolution of the comae. High optical depths along the LOS can be a further challenge. Deconvolving all of these effects is therefore clearly non-trivial. Second, in-situ instruments (e.g. particle detectors, collection plates) provide local measurements – often tens or hundreds of kilometres from the surface. These measurements are not only a product of the surface emission but can be heavily influenced by the dynamics of the gas molecules or dust particles between the surface and the spacecraft. In addition, the location where the measurements were made needs to be taken into consideration. This is due to the fact that the emission and subsequent dynamical effects in the sub-solar direction compared to the one at the terminator or on the anti-solar direction (night side) can be very different. Measurements taken e.g. in a terminator orbit (very common for Rosetta) are likely heavily biased due to the orbit as they are sampling a very specific part of the comae. Both of these fundamental reasons need to be appreciated and dealt with when analysing the data trying to link the comae to the surface, and we will discuss the ways these problems have been tackled.

Before going into more detail we would like to point out a common shortcut that can be taken when trying to link the comae to the surface. In the pursuit to detect surface sources of gas we are faced with the problem that remote sensing instruments that are capable of detecting gas, in particular infrared imagers such as VIRTIS on Rosetta, lack the spatial resolution compared to simultaneous visible imaging to resolve small scale gas coma structures that could be traced to the surface. Scanning instruments such as VIRTIS-H and MIRO can also map the inner gas coma. For such maps, the disentanglement between the spatial and temporal variations can become challenging because it may take hours (a significant fraction of the nucleus rotation of 12.4 h in case of comet 67P (Mottola et al. 2014)) to build up a map. To resolve some of the fine structures of the gas coma a spatial resolution of the order of tens of meters would be desirable. We therefore often use dust as a proxy for the gas because it is easily detected with high resolution visible images such as the ones acquired by OSIRIS on Rosetta. This approach is indeed valid as long as the dust is coupled to the gas which can be safely assumed close to the surface. This assumption does break down for e.g. very dusty outbursts. We will discuss further limitations of this assumption later in the paper. Unfortunately, in the case of 67P the brightness of the dust coma is mostly orders of magnitudes lower than that of the surface and can hence not be seen against the bright backdrop of the illuminated nucleus. There are some cases where the dust brightness is high enough to be detected against the nucleus background, and in these cases the source can be accurately detected. This regrettably is seldom the case. Thus even for this case of linking dust features to the surface a traceback method needs to be applied for most coma structures.

To address the challenges posed above and given the current state of research we will begin in Sect. 2 by describing what we know about the physical processes involved from the surface to a few tens of kilometres above it with respect to the gas and dust emission from cometary nuclei. In Sect. 3 we describe how multidimensional cometary gas and dust models have developed since the Halley encounter of 1986 to today. In Sect. 4 we present the different approaches used and results obtained to link coma data to the surface. We will also discuss the limitations of these approaches.

## The Physics of Developing Cometary Comae

In this section we want to explore the current knowledge of the physical processes that transpire from roughly one metre below the surface of the nucleus to a few tens of kilometres above it. Historically there has been a lot of theoretical work done, and, as computers have emerged, numerical approaches have been developed and become more sophisticated as the computing power has increased over the course of the past decades. In the following we will go through the different stages in natural sequence, from a refractory-ice mixture on the surface that is heated and causes the ice to sublimate, up to the point where the gas molecules and dust particles reach a spacecraft detector tens of kilometres from the surface.

### Illumination, Heating, and Sublimation

We start at the origin of the activity which is the close surface layer composed of a mixture of refractory material and ices. For a detailed description of what we have learned about this layer we refer to Groussin et al. (2019) within this book. For our purposes we are interested how the surface responds to heat input from solar illumination, and it is sufficient that we have learned that cometary nuclei have very low thermal inertia, $\Gamma $ (e.g. Gulkis et al. 2015, Schloerb et al. 2015, Choukroun et al. 2015, Marshall et al. 2018 for 67P, and Groussin et al. 2013 for 9P/Tempel 1). This implies that the diurnal heat wave does not penetrate deep into the interior and thus that sublimation takes place relatively close to the surface if we see the sublimation rate responding fast to illumination changes as shown e.g. by Zakharov et al. (2018a). Furthermore, we assume that the considered surface element is flat i.e. must be understood as an effective surface rather than a real rough surface. For the case of a homogeneous opaque continuous medium the heat balance to be solved for each surface element is thus

1 $$ \frac{S(1-A_{H})}{r_{h}^{2}} \cos (i)\cdot \delta + \Phi _{r}= \epsilon \sigma T^{4} +Z(T) \mathcal{L} + \kappa \frac{dT}{dz}, $$

where on the left side we have the solar energy input which is proportional to the solar constant, $S$, at 1 AU, and the cosine of the solar incidence angle with respect to the surface normal, $i$, and inversely proportional to the square of the heliocentric distance, $r_{h}$, and $A_{H}$ is the bolometric Bond albedo. $\delta $ is a step function which takes the value 1 if a surface element is sunlit and 0 if it is in shadow or on the night side. A further contribution to the energy input comes from the re-radiation flux, $\Phi _{r}$, from other surfaces within the respective field of view of the considered facet. On the right side we have first the thermal re-emission term with the temperature, $T$, to the fourth power, the Stefan-Boltzmann constant, $\sigma $, and emissivity, $\epsilon $, then second the sublimation term with the latent heat, ℒ, and net sublimation flux, $Z(T)$. Knowing the surface temperature, it is possible to calculate the gas production using the classical Hertz-Knudsen formula and the velocity distribution function (VDF; in this case semi-Maxwell distribution) for outgoing molecules. The sublimation rate without back flux is $Z(T) =P(T)/(0.5\pi \,v_{th})$, where $P(T)$ is the water saturation vapor pressure and the average thermal speed $v_{th}(T)=\sqrt{8Tk_{b}/ (\pi m_{g})}$, where $k_{b}$ is the Boltzmann constant and $m_{g}$ is the mass of the molecule. Finally, there is the conduction term with $\frac{dT}{dz}$ being the temperature gradient with depth, $z$, and with the thermal conductivity, $\kappa $, which is related to the thermal inertia, $\Gamma $, defined as

2 $$ \Gamma = \sqrt{\kappa \rho c}, $$

with $\rho $ the effective density and $c$ the specific heat capacity. $\Gamma $ represents the ability of the surface to respond to the temperature forcing provided by the solar illumination, effectively yielding a time lag between the maximum energy input and maximum surface temperature. The case when thermal conductivity is neglected will result in the maximum possible gas production rate (Keller et al. 2015).

Equation (1) thus defines the sublimation temperature and sublimation rate as a functions of the energy input from the Sun and the topographical context of the considered surface area. The complex shape of 67P’s nucleus has shown the need to incorporate shadow casting of parts of the nucleus on large areas. Thus, the direct solar input radiation can be zero, even if $\cos (i) >0$ (when $\delta =0$ in Eq. (1)). This is especially pronounced in concave regions, such as the Hapi region on comet 67P (Thomas et al. 2015c) which can experience two phases of complete darkness per comet rotation during parts of the cometary year.

### The Dust and Ice Boundary Layer

In the previous section we have discussed the process that leads from the illumination of the surface and the associated heat input to the sublimation of ices. Determining the flux of sublimating gas from a bare ice surface is in general not sufficiently accurate. This is due to the fact that very little to no ice has been found directly at the surface (Pommerol et al. 2015; Capaccioni et al. 2015). There are three possibilities to explain this. First, most of the surface is indeed completely devoid of ice. Second, the ice is masked within a mixture of refractories (Yoldi et al. 2015). Third, the ice-containing material is covered by a thin layer of pure refractory material. The first of these three options seems unlikely because the comet appears to be active everywhere, i.e. no completely inactive areas have been discovered. If the second option is valid sublimation takes place at the surface itself. The implications of the third option need to be discussed, considering that a large fraction of the surface of 67P is covered by dust, resulting in smooth looking surfaces, that has likely been redeposited by airfall (Thomas et al. 2015a). In this case the gas from the sublimated ice will flow through a dry dust layer before being released into the coma. During day time, this dry dust layer is potentially much hotter than the sublimating layer because it has no ice cooling it and has thus the potential to alter the gas properties in at least three ways. First, a porous dust layer weakens the gas production rate, and in the most extreme case quenches it completely. Second, the gas can be heated if the molecules interact sufficiently with the dust layer. Third, the VDF can be altered for a flow with or without a dust layer. In general we assume the angular distribution of the emitted gas obeys a cosine law ($\sim \cos ^{n}{i}$), with power, $n$, and angle to the surface normal, $i$. For sublimation from a plane $n=1$ at 1 AU. For more complex cases, $n>1$ can be used as a good approximation. A dust layer through which the gas needs to flow before escaping can thus alter the amount of gas escaping, the temperature of the gas and thus the energy available for acceleration of the gas due to molecular collisions but also the directionality of the molecules upon emission. We will break down the different possible structures of the surface boundary layer in the following sections going from the simplest to the more complex ones.

In the simplest case, it is assumed that solid ice is located on the surface of the cometary nucleus and sublimates directly into vacuum. Then, the ice temperature is determined from a simple algebraic equation expressing the energy balance on the surface (model A in Keller et al. 2015) and Eq. (1) without the last term. In a more accurate approximation, to calculate the surface temperature, it is necessary to solve the heat transfer equation for the surface layer. The estimates made in Keller et al. (2015) show that the difference in gas production estimates between these two approaches to determining the ice surface temperature is insignificant ($\sim10\%$), if the sublimation rate is high enough (that is, at heliocentric distances $<3~\mbox{AU}$).

The first modification of this simplest model is related to the fact that cometary ice (even if it exists on the surface) has, plausibly, considerable porosity. The porosity of the material in the general case leads to the fact that both the process of absorption and release of energy and the process of sublimation acquire a volumetric character, that is, they take place in a certain near-surface layer. Note that, if we neglect the temperature gradient in the layer in question, then porosity does not lead to any new results. Both the rate of sublimation and the VDF of the molecules remain the same as in the model where a solid (i.e. non-porous) slab of ice is considered. It is clear that a layer can be considered as quasi-isothermal only if its thermal conductivity is large. This assumption contradicts the results obtained during the mission (Schloerb et al. 2015; Choukroun et al. 2015). The near-surface layer is obviously non-isothermal, which makes the problem even more difficult. The most complete discussion of a suitable model and results is presented in Davidsson and Skorov (2002) and Davidsson and Skorov (2004). It was shown that in the case of dirty porous ice volumetric effects may lead to a noticeable change in the rate of sublimation. The nature of the VDF for the case of a non-isothermal porous medium has not been studied, but we can assume that the deviation from the semi-Maxwell distribution should be less than for the case when the nucleus surface is covered with a non-volatile porous crust. We now turn to the consideration of the last case.

The results obtained by instruments on board Rosetta (Capaccioni et al. 2015) have shown that the surface of 67P is covered with a non-volatile crust. Ice on the surface appears to be rare. At the same time, there are currently no publications showing the presence of areas on 67P where this crust effectively turns off gas production, i.e., gas activity is observed ubiquitously. This in itself does not yet prove that there are no inert areas but rather that they are difficult or maybe with the current data even impossible to detect because of the rapid lateral expansion of the gas that can hide inert surfaces (see e.g. Marschall et al. 2020a). This could suggests that the dust layer has a high porosity, possibly similar to the macroscopic porosity of the nucleus (Preusker et al. 2017; Kofman et al. 2015; Brouet et al. 2016). The low thermal inertia of the surface layer is also consistent with this conclusion. If gas molecules must pass through a non-isothermal porous crust, then many of the characteristics that are important to us, namely the rate of sublimation (or the density of the gas on the surface), and the VDF of the molecules can change significantly during diffusion.

Considering changes in gas parameters when passing through a porous medium, two cases can be distinguished: (a) the porous medium is described as a system of microchannels (or orifices), that is, the gas flow is regarded as internal with respect to the scattering medium, (b) the porous medium is described as a system of particle-scatters, that is, the gas flow is considered as external to the scattering medium. In both cases, due to collisions and scattering of gas molecules with a non-volatile fraction, the effective sublimation rate is weakened. In addition, temperature and VDF also change.

The analysis of free molecular gas flow through channels has more than a century of history and goes back to the work of Maxwell and Knudsen. Considering the diffuse reflection of molecules from the walls of a cylindrical channel, the latter author obtained a simple relationship in which effective gas production is directly proportional to the radius, $r$, and inversely proportional to the channel length (Knudsen 1909). This formula agrees well with the experimental results only for long tubes (channel length ≫ channel radius). Later, Clausing (1932) proposed a more complex formula that is valid for straight channels of arbitrary length. In order to take into account the fact that the shape of the natural channels differs from a straight cylinder, the so-called phenomenological coefficient of tortuosity $\tau $ ($>1$) is introduced. There are many theoretical and empirical expressions evaluating this parameter (see for example Skorov et al. 2011). However, its specific value cannot be determined without a detailed description of the structure of the porous medium. The Knudsen and Clausing formulas are widely used in theoretical cometary models (capillary models) in order to evaluate the weakening by a porous dust layer.

For the capillary model the angular distribution of the emitted molecules was investigated, for example, by Nanbu (1985) and Skorov and Rickman (1995), using the test-particle Monte Carlo method. They have shown that even for a small layer-thickness to radius ratio ($\sim1$) the distribution shows a marked deviation from the ideal cosine law. For a larger thickness it is collimated near the axis of the orifice. The difference is about 7–9 degrees. For our purposes, this means that the macroscopic velocity of the gas (and therefore, for example, the reactive force causing non-gravitational perturbations in the motion of the comet) exceeds the velocity calculated for sublimation from the plane.

Experiments (Krause et al. 2011) and theoretical models (Kaviany 1995) show that the thermal conductivity of a porous medium can be two to three orders of magnitude lower than the thermal conductivity of a non-porous solid. In the porous case, the temperature difference at the boundary of the dust layer (having a thickness of only a few millimetres) can be tens of degrees (Skorov et al. 2017). When molecules collide with a scattering medium, thermal accommodation occurs, that is, both the velocity direction and its magnitude are modified. A change in the effective gas temperature during passage through a non-isothermal channel was considered in Skorov and Rickman (1995). It was shown that the temperature of the emitted molecules differs markedly from the temperature of the subliming ice already for a channel length of several channel radii.

The Monte Carlo test-particle method can be effectively used also to analyse a free molecular flow through a porous medium, described as a system of particle scatters. This approach allows us to get all the necessary information about the outgoing molecules (e.g. the resulting gas production and VDF). For example, a microphysical computational model for molecular flow in a random porous medium formed by packed spheres was presented and the weakening effect was investigated in detail in Skorov et al. (2011). The main transport characteristics such as the mean free path distribution and the layer permeability were calculated for a wide range of model parameters varying in the range of values expected for the near-surface regions of a cometary nucleus. The results were compared with those obtained by idealized capillary models, and a practical way was suggested to adjust the algebraic Clausing formula taking into consideration the nonlinear dependence of permeability on layer porosity. Although the gas diffusion through a non-isothermal layer has not been considered in this paper (Skorov et al. 2011), the obtained distribution of the mean free path of molecules as a function of porosity makes it possible to estimate the change in gas temperature. Even at very high porosity ($\sim85\%$), the mean free path is shorter than approximately two particle sizes. This means that, like for the capillary model, a substantial part of the molecules changes speed and temperature during transport through the hot non-volatile porous dust layer.

An approach where a random porous medium consisting of spheres is generated via either random ballistic deposition or random sequential packing is rather general. While this approach can provide us with any targeted porosity it does not fully represent real porous medium (rock) samples. Feldkamp et al. (1989) first reported micro-CT X-Ray technology in the 1980’s, and it was used to study bones. Micro-CT technology offers a three-dimensional representation of a rock with high resolution on the scale of μm. It also offers a highly accurate method to measure material porosity. Micro-CT technology attracted interest in the last decade from the petroleum industry for representation of natural gas reservoir porous media (Okabe and Blunt 2004; Dong and Blunt 2009). It is now the state-of-the-art method to represent porous medium structure with a resolution of a few microns.

**Fig. 1:**
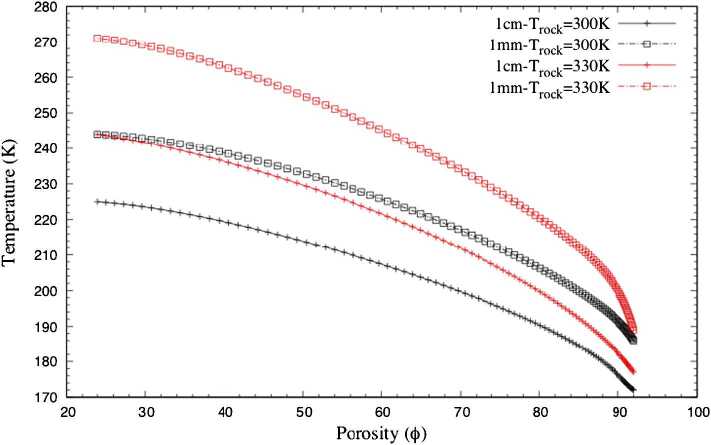
Gas temperature as function of porosity, $\Phi $, at 1 mm and 1 cm above the surface for two rock temperatures, $T_{\text{rock}} = 300~\text{K}$, 330 K. The graphs were obtained by a polynomial interpolation from six different rock samples. Figure adapted from Christou et al. (2018)

**Fig. 2:**
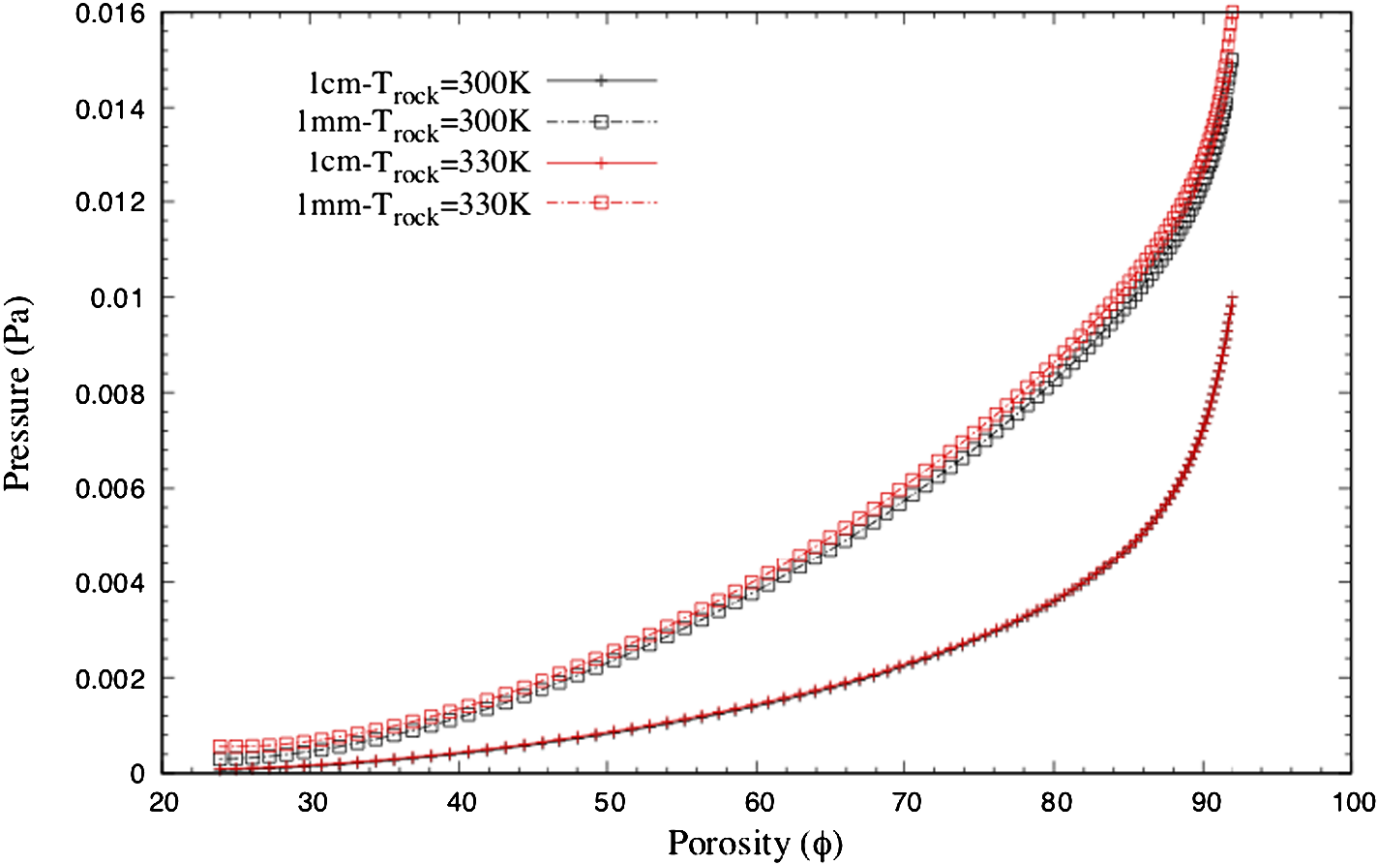
Gas pressure as function of porosity at 1 mm and 1 cm above the surface for two rock temperatures, $T_{\text{rock}} = 300~\text{K}$, 330 K. Figure adapted from Christou et al. (2018)

Christou et al. (2018) and Christou et al. (2019) have directly applied the Direct Simulation Monte Carlo (DSMC) method to the computational mesh obtained from micro-CT images of highly porous rock samples. The flow of sublimating water ice from beneath the sample through the porous layer was studied. At higher porosities, the outgassing temperature remains nearly constant over a longer distance above the surface than at low porosities, $\Phi < 45\%$. Lower porosity media are associated with higher surface gas temperature as the gas interacts more strongly with the warmer surface layer. This may be explained by the fact that, as low porosity implies a low level of void, gas passing through a medium with low porosity is heated more effectively by the higher temperature and expands quickly as it exits. We note that porosity in the range of 88–90% yields a surface gas temperature below 200 K for $T_{\text{rock}} = 300~\text{K}$ and just above 200 K in the case of $T_{\text{rock}} = 330~\text{K}$. Gas passing through high-porosity media receives less heating and experiences an expansion and therefore cooling before exiting the medium. Similar results were reported by Skorov et al. (2011). This is illustrated in Fig. 1. As the porosity increases the gas temperature decreases. Furthermore, Fig. 2 shows the surface pressure as function of porosity. Higher porosity leads to higher pressure. Porosity can thus play an important role in altering gas properties before emission at the surface. Even quenching of the activity can be achieved with a dust layer of very low porosity. Christou et al. (2019) have shown that due to the large variations of observed gas pressures as a function of porosity, lateral gradients in porosity along the surface can produce strong lateral gas flows. Coma inhomogenities can be attributed to non-uniformly distributed sources of H_2_O and CO_2_ over the surface of the nucleus (Fink et al. 2016) or variations in illumination in conjunction with the local topography (Marschall et al. 2016). These lateral flows caused by lateral porosity gradients may be capable of generating dust grain transports. Lara et al. (2015) reported that dust particles on the scale of mm could be ejected. More recently, Lai et al. (2016) calculated for 67P that during peak production, near perihelion passage, grains as large as 10 mm can be ejected. Furthermore, dust particles up to the size of decimetres have been observed flying around 67P (Agarwal et al. 2016). Based on those observations and the lateral velocities predicted by Christou et al. (2019) we can conclude that porosity gradients can generate the necessary lateral flow to transport dust grains after they are ejected from the surface.

These considerations above have covered what may be referred to as the contiguous activity. In addition, short-term activity (“outbursts”) has been observed on various time and spatial scales throughout the Rosetta mission (Tubiana et al. 2015; Vincent et al. 2016b; Lin et al. 2017; Bockelée-Morvan et al. 2017; Rinaldi et al. 2018). Outbursts occur not only in case of 67P but are typical for comets. While some of these events can be associated with the erosion of cliffs (Vincent et al. 2016c; Pajola et al. 2017), others were likely driven by either the sublimation of freshly uncovered supervolatiles (Skorov et al. 2016b) or by the release of energy stored in the cometary subsurface (Knollenberg et al. 2016; Agarwal et al. 2017).

### Expansion of the Gas

**Fig. 3:**
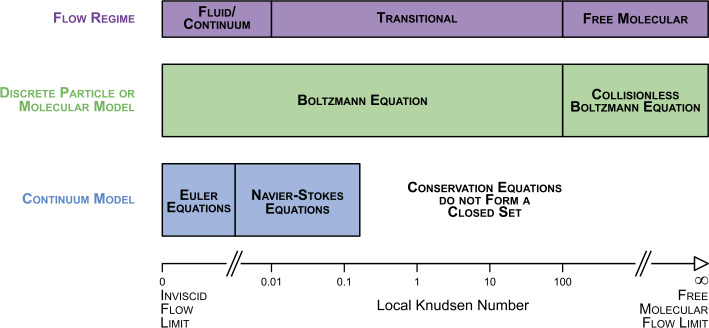
Different flow regimes as a function of the Knudsen number as well as the intervals for which different models (continuum and discrete) are valid. This figure has been adapted from its original in Bird (1994)

The gas flow in the inner coma of an active cometary nucleus offers severe challenges for numerical simulations because of its complex spatial structure. A macro-scale simulation involves consideration of the complex nucleus shape and topography while a micro-scale simulation will involve a detailed local near surface outgassing modelling. In the most general case, the flow in the coma is a juxtaposition of regions with widely differing conditions – from fully collision-less to fluid. The degree of rarefaction is characterised by the Knudsen number $Kn$:

3 $$ Kn=\lambda /L, $$

where $\lambda $ is the mean free path of the molecules (MFP) and $L$ is the characteristic dimension. To describe the complete flow by a single overall Knudsen number a characteristic scale of the flow (traditionally the equivalent radius of the nucleus) can be used as $L$. To characterise the rarefaction of the flow locally, the scale length of the macroscopic gradient can be used as $L$ (e.g. using the gas density, $n_{g}$, such that $L = n_{g}/|\nabla n_{g}|$). Depending on the local $Kn$ three flow regimes can be roughly distinguished (illustrated in Fig. 3):

1. continuum/fluid, $Kn<0.01$;
2. transitional, $0.01 \leq Kn \leq 100$;
3. free molecular, $Kn>100$.

As was shown in the previous sections, the gas coma is formed by the ice sublimation from the nucleus in different ways. The VDF of the sublimating molecules is non-Maxwellian (it is commonly assumed to be half-Maxwellian corresponding to the surface temperature). Therefore, a non-equilibrium layer (Knudsen layer) always occurs adjacent to the surface. Due to inter-molecular collisions the initial VDF evolves (relaxes) and may become at some distance (if and when there are a sufficient number of collisions) completely (or quasi) Maxwellian. The expansion of the flow leads to a decrease of collisions with radial distance and therefore, even if an equilibrium flow was established at the top of the Knudsen layer, the flow becomes non-equilibrium again at larger distances due to an insufficient collision rate to maintain the equilibrium distribution.

The structure of the flow depends considerably on the rarefaction of the flow. For high initial densities of the gas (i.e. high production rates of the surface) three characteristic regions can be distinguished in the flow:

1. subsonic near-surface layer;
2. supersonic continuum flow;
3. supersonic non-continuum, non-equilibrium flow.

The boundary between regions (1) and (2) is the surface where the gas velocity is equal to the local sound velocity. The boundary between regions (2) and (3) is the conditional boundary of the region of the continuum flow (its exact position depends on the formal definition of the continuum breakdown). The part of the subsonic region near the surface is also in non-equilibrium (owing to the Knudsen layer). The thickness of the Knudsen layer depends on the specific conditions, but in general, it is about 10–100 mean free paths (Ytrehus 1975; Davidsson 2008). When $Kn$ decreases, it leads to a decrease of the non-equilibrium subsonic layer and an increase of the continuum supersonic flow (the boundary of the continuous flow is shifted downstream). When $Kn$ increases, the subsonic and the Knudsen layers become thick, and at some rarefaction the Knudsen layer becomes thicker than the subsonic layer. The increase of $Kn$ leads to a decrease of the supersonic continuum flow region because the outer boundary of the continuum flow approaches the surface. At some rarefaction the near-surface non-equilibrium layer (Knudsen layer) and the outer region of non-equilibrium flow merge together, and the Knudsen layer as a relaxation zone of the initial non-equilibrium cannot be distinguished from the outer non-equilibrium region.

The interactions of supersonic fluxes in the coma lead to the appearance of shock structures (with localised enhancements of density, temperature and decrease of velocity). The rarefaction of the flow with such structures is characterised using the scale length of the macroscopic gradients. Therefore, even if the overall flow is dense, locally the flow may be rarefied and non-equilibrium.

In general, the transfer of thermal energy into kinetic energy in a rarefied flow is less efficient than in a fluid flow, therefore the rarefied flow accelerates slower, and the flow structures are more diffused.

The gas dynamics approach uses for its description of the gas only the first moments of the VDF – the number density, bulk velocity, and temperature (Euler equations, see Fig. 3), heat conduction, and viscous dissipation (Navier-Stokes equations, see Fig. 3). This approach is appropriate for those regions where the VDF is either a strict Maxwellian (i.e. the fluid flow) or a moderately distorted Maxwellian. Since this approach can’t be used for the simulation in the Knudsen layer, it is necessary to introduce an additional model which links parameters on the surface and on the top of the Knudsen layer – the initial boundary for the gas dynamics simulations. It was shown that the gas dynamics approach with appropriate boundary conditions can provide a physically adequate description of the gas flow even at a moderate degree of rarefaction, but in this case, it is necessary to verify the adequacy of solutions in each particular case (Crifo et al. 2002a, 2003; Lukyanov et al. 2005; Zakharov et al. 2008). It is important to note the difference between “an adequate description” and “a precise description”. For example, for the expansion into a vacuum at large distances from the source the Euler solution predicts a continuous acceleration of the gas but the Navier-Stokes solution predicts deceleration due to the excessive viscous dissipation. In this case, the solution of the Euler equations is physically adequate (the gas does accelerate in reality) but it is not precise (e.g. it exaggerates the transfer of thermal energy into kinetic energy). The solution of the Navier-Stokes equations, on the other hand, is physically inadequate. In many cases, the fluid approach keeps physical adequacy of the rarefied flow description – though with different degrees of precision. It is thus necessary to verify the precision for every case.

Gas kinetics methods – based on the collisional Boltzmann equation (see Fig. 3) or the direct simulation Monte Carlo (DSMC) method proposed by Bird (Bird 1994) – are appropriate at any level of distortion of the VDF. The main drawback of these methods is their high computational demands. With the emergence of high-performance computational facilities, the DSMC method became the main method of simulations for rarefied non-equilibrium flows. For example, DSMC was used for simulation of the coma in e.g. Combi (1996), Skorov and Rickman (1999), Crifo et al. (2002a, 2003), Tenishev et al. (2008), Davidsson (2008), Zakharov et al. (2009), Fougere et al. (2013), Finklenburg (2014), Marschall et al. (2016).

As the rate of collisions (if there were any initially) between molecules decreases with distance the momentum and energy exchange decreases as well. This results in the conservation of the flow velocity and internal energy with distance. Also, the kinetic temperature (as a measure of the velocity dispersion with respect to the mean velocity at some point) freezes for the parallel component and slowly decreases for the perpendicular component. Therefore, starting from some distance the gas flow approaches the flow with practically constant temperature and velocity (directed radially from the centre of the nucleus), and the variation of the density, $n_{g}$, as a function of distance to the comet, $r$, changes according to the “inverse $r^{2}$” law:

4 $$ n_{g} \propto \frac{1}{r^{2}}. $$

The “Haser model” (Haser 1957) uses this property of the flow and includes additionally photo-chemical destruction of molecules. This model is still the most commonly used approximation owing to its apparent simplicity. But it should be noted that it is physically adequate only at a large distance from the nucleus.

Depending on the size of the simulation region the relative importance of physical processes varies. Usually, in the near-nucleus region the collisional processes dominate over photo-dissociation and ionisation, but for large distances and thus long travel times of the molecules the time scales of photo-dissociation and ionisation may become significant (Crovisier 1989; Xie and Mumma 1996; Combi et al. 2004). For the typical conditions, the velocity of gas molecules is of the order of hundreds of meters per second and the typical time scale of processes such as photo-dissociation and ionisation is about $10^{5}$–$10^{7}$ seconds. For regions greater than $10^{4}~\text{km}$ it is, therefore, necessary to take into account the processes of photo-dissociation and ionisation. The numerical simulations in a large region can be complicated additionally if the settling time of the flow (which is proportional to the size of the region) is longer than the characteristic variation time of boundary conditions (e.g. due to the rotation of the nucleus). In this case, the flow might never reach a steady state.

### The Dust Activity Paradox

Up to this point we have mainly focused on the icy constituents and not on the refractory ones. When dust is separated from the surface it can be accelerated in the gas flow. We will discuss this in more detail in the next section. Very little is known about the refractory and ice mixture at the surface (see also Choukroun et al. 2020, in this book), and thus the nature of how dust is released out of this matrix remains an open problem often referred to as the “activity paradox” (Kührt and Keller 1994). The reason for this paradox is the fact that we see the strong cometary dust and gas activity but do not have a working model of how dust is released. The simplest mechanism where the gas pressure of the sublimating ice overcomes the cohesive forces of the material does not work (Skorov et al. 2017). The material strength inferred for cometary material (e.g. Attree et al. 2018) is much larger than the corresponding gas pressures available. For more details and ways out of this paradox as well as our state of knowledge about the physical properties of the surface we refer to Vincent et al. (2019) as well as Groussin et al. (2019) in this book.

### The Dust Dynamics in the Coma

**Fig. 4:**
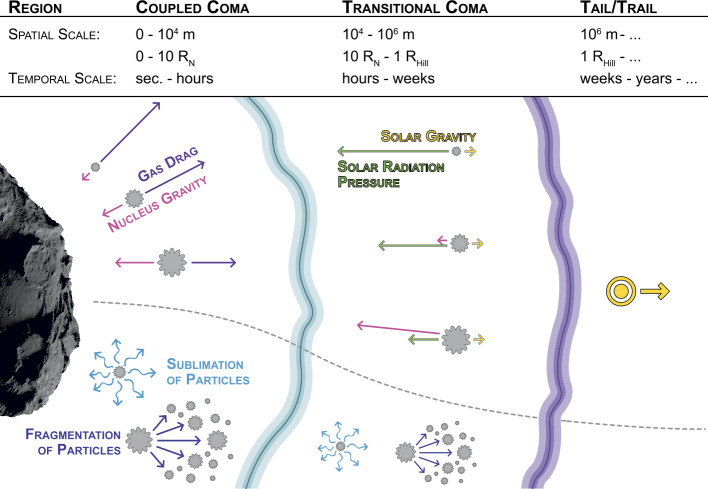
Sketch of the different dynamical regions of the cometary dust environment which we can divide into three regions: (1) The coupled coma region where the dust dynamics is dominated by local forces connected to the nucleus (gas drag and nucleus gravity): (2) the transitional coma region where the dust has decoupled from the gas and small particles transition to being dominated by solar forces (gravity and radiation pressure) and large particles are bound in the gravitational field of the nucleus; (3) the dust tail and trail within which the escaping dust particles are purely governed by solar forces. The boundaries between these regions are complex 3D surfaces and not spherical shells and the spatial and temporal scales are rough estimates for a 67P-like comet at 1 AU). $R_{N}$ is the nucleus radius, and $R_{Hill}$ the Hill radius. See text in this section and Sect. 2.6, 2.8, and 2.7 for more details

Neglecting for the time being the problems associated with ejecting dust from the surface as described in the previous section we can nevertheless continue and discuss the dynamics of the dust particles once they are no longer attached to the surface. At that point dust particles are subject to several forces driving their motion. Figure 4 shows a sketch of the different dynamical regions of the cometary dust environment which we can divide into three regions (in the order from closest to furthest from the surface):

1. **The coupled coma region:** In this part of the coma the dynamics of dust particles is dominated by local forces that are linked to the nucleus. These are the gas drag force and the gravitational force of the nucleus. Because of the non-sphericity of the nucleus the gravitational force from the nucleus is in general non-radial. Likewise because of the complex nucleus shape, local topography, and emission inhomogeneities the gas flow is in general strongly non-radial (beyond the non-radiality that can occur even for a spherical nucleus). Both forces converge to radial forces as the distance to the surface increases.
  The size of this first region extends to from the surface to roughly 10 nucleus radii, $R_{N}$, and the time particles spend in this region is of the order of seconds to hours depending on the dust size.
2. **The transitional coma region:** In this part of the coma the governing forces dominating the dust dynamics transition from local forces linked to the nucleus to solar forces such as solar gravity and solar radiation pressure. In this region, the gas and dust have completely decoupled. This is also the region within which the dynamics of very large particles is dominated by the nucleus gravity. Therefore, this region also encompasses the region within which a separation of the coma occurs: small particles escape the gravitational field of the comet and are dominated by solar forces, and large particles remain bound to the comet and eventually re-deposit on the surface.
  The size of this second region extends roughly to the comet’s Hill radii, $R_{Hill}$, and the time particles spend in this region is of the order of hours to weeks depending on the dust size.
3. **The dust tail and trail:** In this final region of a comet’s dust environment the dust particles have left all influence of the nucleus and their dynamics is solely governed by solar forces (gravity and radiation pressure).

Additional processes to the ones named above can alter the dynamics of the dust particles in all three regions of the cometary dust environment. These processes are related to the properties of the dust particles themselves such as sublimation of icy components of the dust particles or fragmentation. See Sect. 2.8 for more details on these processes. We should stress that the picture described above is overly simplified and that each of these regions has a complex 3D structure. Further, the interfaces between these regions are not sharp spherical shells but rather complex 3D surfaces that vary depending on the dust sizes, the heliocentric distance of the comet, overall activity and its distribution on the surface, and more.

Here we focus primarily on the coupled coma region but will comment on some of the above mentioned additional forces and processes in Sects. 2.6, 2.7, and 2.8.

The accelerating force for a spherical dust particle away from the surface is the gas drag (a non-spherical particle will have additional aerodynamic forces). In the immediate vicinity the nucleus gravity is the main opposing force. For longer time scales solar radiation pressure needs to be included as well but can be neglected in the innermost coma (Tenishev et al. 2011; Skorov et al. 2016a, 2018). The dust can be treated either in a test particle approach within the gas flow or as a separate fluid within a gas-dust multi-fluid approach (e.g. Shou et al. 2017). We will discuss the former here in more detail as it is the more common approach used. In this approach it is assumed that dust does not affect the gas flow. In addition, since in most cases the mean free path of the molecules is much larger than the dust particles size, the free molecular aerodynamics is applied. For this approach (Finson and Probstein 1968; Gombosi et al. 1985, 1986; Gombosi 1987; Sengers et al. 2014; Marschall et al. 2016) the equation of motion in the inertial frame for each dust particle is

5 $$ \begin{aligned} m_{d} \frac{d^{2} \mathbf {x}}{dt^{2}} &= \mathbf {F}_{G} + \mathbf {F}_{D}\\ &= \mathbf {F}_{G} + \frac{1}{2} C_{D} m_{g} n_{g} \sigma _{d} \left | \mathbf {v}_{g} - \mathbf {v}_{d} \right | \left ( \mathbf {v}_{g} - \mathbf {v}_{d} \right ), \end{aligned} $$

where $m_{d}$ is the dust particle mass, and $\sigma _{d}$ the geometric cross-section. The equation evaluates the dynamic properties of the dust at position $\mathbf {x}$ and with velocity $\mathbf {v}_{d} = \frac{d\mathbf {x}}{dt}$, $\mathbf {F}_{G}$ is the gravitational force between the dust particle and the nucleus, $m_{g}$ the mass of the gas molecule considered (in our case molecular water), and $n_{g}$ and $\mathbf {v}_{g}$ are the number density and macroscopic velocity of the gas, respectively. If we assume an equilibrium gas flow and that the mean free path of the molecules is much larger than the dust size, the drag coefficient $C_{D}$ (Bird 1994) is defined as

6 $$ C_{D} = \frac{2 \zeta ^{2} + 1}{\sqrt{\pi } \zeta ^{3}} e^{-\zeta ^{2}} + \frac{4 \zeta ^{4} + 4 \zeta ^{2} - 1}{2 \zeta ^{4}} \text{erf}( \zeta ) + \frac{2 \left ( 1 - \varepsilon \right ) \sqrt{\pi }}{3 \zeta } \sqrt{ \frac{T_{d}}{T_{g}}}, $$

with the gas temperature $T_{g}$, the dust particle temperature $T_{d}$, and $\varepsilon $ is the fraction of specular reflection, and

7 $$ \zeta = \frac{ \left | \mathbf {v}_{g} - \mathbf {v}_{d} \right | }{\sqrt{\frac{2k_{b} T_{g}}{m_{g}}}} . $$

Although we often approximate the dust grains as test particles and consider them to be spherical, especially the larger dust grains are porous and fluffy aggregates (Kolokolova and Kimura 2010; Schulz et al. 2015; Rotundi et al. 2015; Langevin et al. 2016; Bentley et al. 2016; Mannel et al. 2016; Levasseur-Regourd et al. 2018). This affects the dynamics of the particles as shown by Skorov et al. (2016a, 2018) who have found that porous aggregates are accelerated to significantly higher speeds than their compact counterparts. Additionally, rotating particles (Fulle et al. 2015) that have oblate or prolate shapes will also affect the dynamics as studied by Ivanovski et al. (2017a,b). These latter authors have shown that not only may non-spherical particles begin to rotate in the gas flow but they may also accelerate to higher speeds than spherical ones.

### Further Forces Acting on Dust

We have already mentioned the two other forces that influence the dynamics of the dust particles namely solar radiation pressure and gravity. The effect of solar radiation pressure has long been known. Even though dust particles are ejected mainly in the sunward direction cometary tails point in the anti-sunward direction. This is caused by solar radiation pressure decelerating the dust particles as they move towards the Sun until they come to rest and are then pushed in the anti-sunward direction. This back flux towards the comet has, in case of comet 67P, even been found close to the nucleus by GIADA (Colangeli et al. 2007) on board Rosetta.

The effect of gravity is twofold. First, it is an additional opposing force when particles are lifted and thus even in absence of any cohesive forces provide an upper size limit that can be ejected from the surface. Second, large particles which are not accelerated by the gas beyond the escape speed will go into orbit or ballistic trajectories. In the case of 67P its complex nucleus shape leads to a complex gravity field and thus to a non-trivial gravitational influence. Dust deposits from gravitational back-fall on the surface alter the surface properties which in turn influence the nature of activity. In the case of 67P this contributes to the observed seasonality of the activity.

**Fig. 5:**
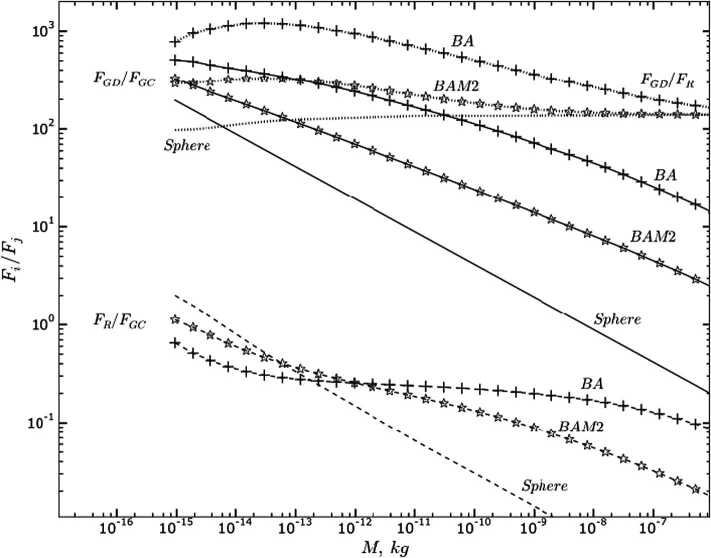
Ratios of drag force, $F_{GD}$, gravity, $F_{GC}$, and radiation pressure, $F_{R}$, on dust particles of different masses: $F_{GD}$/$F_{GC}$ (solid curves), $F_{GD}$/$F_{R}$ (dotted curves), and $F_{R}$/$F_{GC}$ (dashed curves) as functions of aggregate mass at 2 km from the comet centre. Results for ballistic aggregates (BA, crosses) and ballistic aggregation with monomer migration (BAM, stars) and non-porous spheres are shown. Euler flow was assumed for the gas flow with a production rate of $2~\mbox{kg}\,\mbox{s}^{-1}$ from a spherical nucleus of 2 km radius. Figure adapted from Skorov et al. (2018)

Figure 5 illustrates the ratios of the three major forces acting on a dust particle (Skorov et al. 2018). Different types of particles are shown, from the common assumption of solid spheres to ballistic aggregates (hit and stick of monomers; BA) and ballistic aggregates with monomer migration (hit, stick, and roll; BAM). BA have the highest porosity and the solid spheres per definition the lowest. The figure shows that close to the surface the drag force is the dominant force (by orders of magnitude) for the entire mass range of dust particles. The higher the porosity of the particles the higher also the ratio of the drag to the gravitational force. This is mainly driven by the higher cross-section to mass ratio of the porous particles. Radiation pressure is between two and three orders of magnitude weaker than the gas drag interaction for the given dust mass range and does not depend as strongly on mass as the drag-to-gravity force ratio. For spherical expansion of the gas the drag force remains dominant over gravity even at 20 km from the nucleus centre (Skorov et al. 2018), but radiation pressure becomes more important, and the respective ratio to the drag force can be close to unity. The authors conclude further for the described case that the drag force is indeed dominant for the near-nucleus environment ($<20~\text{km}$) but that beyond that radiation pressure can be a significant factor in the dust dynamics at distances as close as 100 km from the nucleus centre. Finally, the shown results also imply that the assumption of solid spheres leads to the lowest acceleration and thus lowest terminal dust speeds. The higher the porosity the higher the acceleration and thus the higher the terminal dust speed. The solid sphere approximation thus provides for a strict lower boundary for the dust speed.

### Redeposition of Dust on the Surface, and Re-Condensation of Gas

So far we have primarily described the mechanisms of mass loss of the comet. But Rosetta has shown that there are two important processes of redeposition on the nucleus which were not well appreciated pre-Rosetta. The first one has been hinted at in the previous section. Due to gravity dust particles – or rather large chunks – might not reach escape speed and are therefore redeposited. This is mainly evident in the morphology of the nucleus surface (Thomas et al. 2015c; El-Maarry et al. 2015, 2016, 2017) showing widespread dusty deposits in particular in the northern hemisphere. This has been described in more detail in Thomas et al. (2015b) who have also shown how particles are gravitationally bound and redeposited. The deposition of particles, especially in gravitational lows, has become quite clear since then. This has been further studied by Lai et al. (2016) who have found that gravitational lows are indeed primary deposition areas. The idea of a seasonal mass transport from the southern to the northern hemisphere has also been pointed out by Keller et al. (2017). This seasonal dust transport can lead to significant altering of the surface properties which in turn define the respective activity. Activity is therefore not simply a function of the local surface properties but can be influenced by non-local long term alterations due to mass transport.

Metre-sized chunks of debris falling back towards the surface have been directly observed in at least one image sequence obtained at 2.6 AU, when comet 67P was outbound from perihelion (Agarwal et al. 2016). Due to the nucleus rotation and the varying trajectories of individual chunks, the material will get distributed widely across the comet (Keller et al. 2017). Generally, the locations of sources and sinks of fall-back debris are determined by the local gas pressure, which in turn is likely mainly determined by solar irradiation and changes with season (Lai et al. 2016; Pajola et al. 2017).

The other possible deposition is that of re-condensation of water vapour. As found by De Sanctis et al. (2015), Filacchione et al. (2016a) a diurnal frost layer has been found on the surface of comet 67P. This surface ice can either be due to re-condensation from below in the near-surface layer due to a temperature inversion caused by thermal lag, or due to re-condensation of gas in the coma from above the surface. Liao et al. (2018) have shown that this second process is also feasible.

Different authors (Fornasier et al. 2016; Filacchione et al. 2016b; Ciarniello et al. 2016; Fornasier et al. 2017) found an increased surface ice content in the southern hemisphere near perihelion, indicating temporary removal or thinning of an otherwise present dust mantle, and the exposure of relatively pristine icy material. These effects were accompanied by significant surface changes such as boulder displacements and vanishing of structures (Fornasier et al. 2019).

### Additional Processes Affecting Dust and Gas Flows

Now that we have described the dynamics that govern dust particles, we will also highlight the regime within which this is applicable. There are two main restrictions to the approach described in the previous sections. First, if the flow has a high dust-to-gas mass ratio (mass loading) the two flows cannot be treated independently. Because the dust is accelerated by the gas it draws energy out of it. When there is enough dust in the flow that this ratio becomes significant then the presence of the dust will influence the gas flow. On the one hand the gas will be heated by the usually much hotter dust particles, and on the other hand the dust will decrease the energy in the gas flow and thus tends to slow it down. Additionally, if there is a lot of dust – which is heated very efficiently – it will thermally re-radiate energy that can reach the surface and be an additional source of energy into the surface. The approach above also excludes any icy component in the dust that can sublimate. If ice-bearing dust particles or debris are able to sustain a temperature gradient, e.g. between their illuminated and shadowed sides, the recoil force from asymmetric outgassing can affect their dynamics (Kelley et al. 2013; Agarwal et al. 2016; Güttler et al. 2017). This would also be an additional gas source term – a so called extended source – above the surface and thus alter the gas flow. For comet 67P there has not been any direct evidence for such an extended source but it has been observed for other comets. This process requires coupling of the gas and dust flows and thus a simultaneous numerical treatment of the gas and dust comae. Apart from sublimation, dust particles can also fragment e.g. due to collisions, thermal stresses, or sublimation of icy components. This will alter the size distribution of the dust flow. Further, particles can be charged by the solar wind and their dynamics strongly influenced by electromagnetic forces. This has been first studied by Horanyi and Mendis (1985) and Wallis and Hassan (1983). The latter have found that wavy dust features far down the tail could be explained by charged dust. The charging of dust mainly alters the dynamics of sub-micron to micron particles and acts on long time scales.

### Observations of the Dynamics of Dust from the Surface into the Coma

**Fig. 6:**
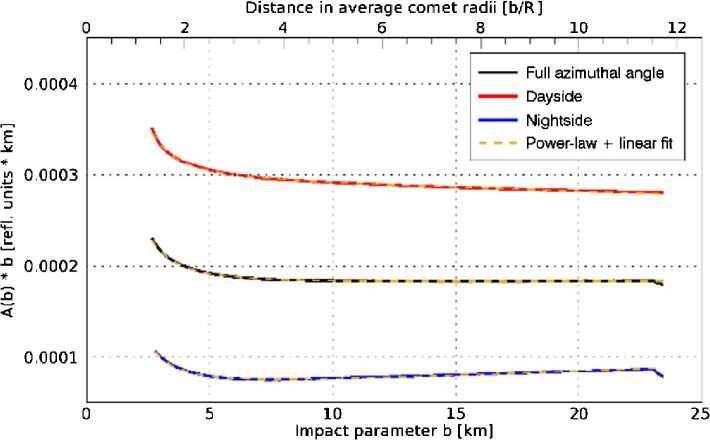
Brightness, $A$, for the case of 67P multiplied by the distance, b, as a function of the distance (taken from Gerig et al. 2018). The black curve shows the full result and the red/blue curves are restricted to the day/night side of the comet

We expect the density of a flow that is radial and collisionless to drop with the inverse square of the distance to the nucleus centre. Similarly to the gas density (Eq. (4)), assuming such a force-free radial outflow, the dust density $n_{d}$, for a specific dust size, as a function of distance $r$ can be calculated as

8 $$ n_{d} = \frac{Q_{d}}{4\pi r^{2} \,m_{d} \,v_{d}}, $$

with $Q_{d}$ being the total dust mass production rate, $m_{d}$ the dust particle mass, and $v_{d}$ the constant outflow velocity of the dust particles. However, imaging instruments like OSIRIS on Rosetta do not see the density but rather the dust brightness which is the result of the column-integrated density (column density) along the LOS convolved with the scattering properties of the dust particles. In an optically thin medium the observed brightness is proportional to the column density. Due to the integration along the LOS Eq. (8) implies that for a free radial flow the column density should drop by the inverse of the distance. Because the brightness of the dust is proportional to the column density, the brightness multiplied by the distance should thus a conserved quantity as a function of distance. Thomas and Keller (1990) introduced this quantity as the so-called “azimuthal average” in a study about the dynamics in the inner coma of comet 1P/Halley. They used aperture photometry to study the change in brightness as a function of distance to the comet centre of mass. Deviations in the azimuthal average from this conservation law contain information about additional physical processes involved in the inner coma dynamics compared to free radial outflow. Different processes such as acceleration or fragmentation of the dust particles have different impacts on the azimuthal average. Several of the processes discussed in the literature and their effects on the azimuthal average are revisited below. For one, acceleration of the particles due to gas drag will cause the azimuthal average to drop as a function of distance. This is because the scattering area and thus the brightness of the observed dust disperses into space more quickly than is expected for a free radial outflow due to the increasing velocity (Eq. (8)). When considering fragmentation of dust particles, two different outcomes are possible. On the one hand, for dust particles fragmenting into optically more active daughter particles and thus increasing the scattering area, the azimuthal average will increase with distance from the nucleus (Konno et al. 1993). On the other hand, when a majority of the available dust particles is fragmenting into optically small particles (daughter particles invisible for scattering), the azimuthal average will decrease with distance from the nucleus surface. Sublimation of icy grains in the coma has much the same effect on the azimuthal average like this latter case because here scattering area is lost in the sublimation process. Conversely, optical depth effects can increase the azimuthal average as a function of distance as a larger and larger fraction of the scattering cross section comes into view. The effects of outgassing from localised sources on an extended surface area as modelled by Huebner et al. (1988) and Reitsema et al. (1989) can lead to an increase in azimuthal average with distance from the nucleus. Furthermore, gravitationally bound particles in Keplerian orbits out to the Hill radius can lead to an increase in azimuthal average with distance from the nucleus surface, whereas gravitationally bound particles on ballistic trajectories have the opposite effect as demonstrated by Gerig et al. (2018). In the case of 1P/Halley an increasing azimuthal average curve was found close to the nucleus ($< 100~\text{km}$ or 10 nucleus radii) (Thomas and Keller 1990). In several studies a combination of localised sources over an extended active area and particle fragmentation were identified as the dominating processes in the innermost dust coma of 1P/Halley (Huebner et al. 1988; Szego et al. 1989; Thomas et al. 1988; Reitsema et al. 1989; Thomas and Keller 1990; Konno et al. 1993). At larger distances (100–600 km), the profile was flat as expected for a free-radial flow. In contrast, at 67P a decline in the azimuthal average profile as a function of distance was found, indicating that we are most likely seeing the acceleration of the particles. Using models described in Sect. 4.2, Gerig et al. (2018) demonstrated that this trend can be reproduced in a model only taking into account acceleration of the dust particles entrained in the gas outflow and gravity from the nucleus. They also concluded that other processes in the inner coma such as particle sublimation or contributions from gravitationally bound particles might be necessary to fully explain the data. In a study of the radial profile of a jet-like feature above the Imhotep region around spring equinox 2015, Gicquel et al. (2016) concluded that sublimation of small icy grains is needed to explain the observed steep profile decline in the first 10 km above the nucleus surface. Like for comet Halley, the azimuthal average curve for 67P also flattens out at larger distances from the nucleus ($>12$ km or 6 nucleus radii; Gerig et al. 2018), and the flow can be treated as a force-free radial outflow beyond that distance. Figure 6 shows a typical example of the drop and then flattening of the curve (in black) with distance. Generalised theoretical considerations as shown by Zakharov et al. (2018b) predict the dust to reach 90% of its terminal velocity at a distance of 6 nucleus radii. This is in remarkable agreement with the results for 67P, and also agrees well with the results found for 1P/Halley. During the time of peak activity, about one month after perihelion, Boehnhardt et al. (2016) in ground-based images of 67P found radial brightness profiles flatter than expected for a steady-state coma, which they interpreted as a possible sign of dust fragmentation at nucleus distances $>5000~\mbox{km}$ from the nucleus. Also ground based polarimetric measurements from several months after perihelion hint at the presence of fragmenting dust in the outer coma (Rosenbush et al. 2017). This illustrates how the combination of theory, complex models, and data analysis have become very successful in the understanding of the dynamics within a cometary coma.

## The Development of Multidimensional Cometary Gas and Dust Models

Since the observations of comet 1P/Halley from a close distance in 1986, it has become evident that the near-nucleus coma has a complex spatial structure (previous observations from large distances just suspected that). Therefore, the simulation of such flows requires multidimensional models of the dusty gas flows. In this section, we will briefly describe the development of such multidimensional models which, triggered by the lead up to the encounter of comet 1P/Halley in 1986, have progressed to this day and applied to Rosetta’s data of comet 67P. Though these models build on earlier descriptions in one dimension we will not go into the details of those models.

The first multidimensional models (axially-symmetric and 3D) of a dusty gas coma with the physically consistent description of a flow as a fluid were presented in Kitamura (1986, 1987, 1990), and Korosmezey and Gombosi (1990). These models were based on the numerical solution of the coupled hydrodynamic equations (representing the mass, momentum, and energy conservation). The gas (pure H_2_O) was treated as viscous in Kitamura (1986, 1987) or inviscid in Kitamura (1990), and Korosmezey and Gombosi (1990). The dust was treated as one of the components of the fluid consisting of single-sized spherical grains ($<1~\upmu \text{m}$). The studies were performed for a spherical nucleus with (1) an active spot on an inactive surface (i.e. an isolated jet), (2) a spot of enhanced activity on an active surface (i.e. a jet expanding in a co-current flow), and (3) several active spots. The Knudsen number in the jet near the surface was $\sim 10^{-3}$ (estimated by the radius of the nucleus).

These studies showed the formation of the shock structures due to interactions of several jets and that even a single pure gas jet expanding in a co-current flow produces a shock structure. It was shown that the dust structures are more pronounced and persist even at large radial distances.

In all these works the boundary conditions were postulated in a simplified manner (i.e. without a thermo-physical model of the surface) – a given variation of surface flux or stagnation density and constant surface temperature.

These same problems were studied by Itkin et al. (1995), Crifo et al. (1995), Rodionov (1996) with a more efficient computational scheme. These studies revealed that the “single fluid” approach (when all the dust ejected from the nucleus is treated as one single fluid) leads to an unphysical result (artificially strong enhancement of the density) in the region where dust fluxes penetrate each other. To resolve this problem a “multi-fluid” approach, where the dust flow is represented by several fluids originating from different parts of the surface, was applied. Though this model is physically more accurate it is also computationally more expensive.

The studies by Crifo and Rodionov (1997a), Fulle et al. (1999), and Crifo and Rodionov (1999) included a non-spherical shape of the nucleus. A homogeneous “bean” shaped nucleus was used as a model of comet 1P/Halley in Crifo and Rodionov (1997a) and of comet 46P/Wirtanen in Fulle et al. (1999), and Crifo and Rodionov (1999). Also, a realistic model of surface ice sublimation which takes into account the influence of the ambient flow (see Crifo and Rodionov 1997b) was used to get the gas production of the surface. This model was based on the analytical expression for the Knudsen layer obtained by Cercignani (1981). These studies showed for the first time that even in the case of a homogeneous nucleus, the fluxes emanating from different parts of the nucleus can interact and form shock structures i.e. even the absence of any surface inhomogeneity can result in the appearance of inhomogeneity in the cometary coma.

The effect of a realistic shape for comet 1P/Halley – described by spherical harmonics as derived from observational data of Giotto, Vega-1 and Vega-2 – was studied in Crifo et al. (2002c). This study was performed for cases of homogeneous and inhomogeneous (set of spots) surface properties. This study showed that a more detailed description of the shape leads to a considerably more complicated flow structure near the surface. Most notably, geometrical effects of the surface can be stronger than the effect of a surface inhomogeneity.

A model with two gas components – water and CO – was studied in Crifo et al. (2004). It was assumed that the water sublimation at the surface and the CO diffusion from the interior occur simultaneously. This model was used before Rosetta had arrived to study different plausible conditions in the coma of comet 67P at 3 AU. An analytical “starfish” shape with topological similarity to the shape derived from Hubble Space Telescope observations in 2002 was used for the nucleus. This study showed once again that the coma around a non-spherical nucleus is strongly structured by the topography.

Ground-based observations have a large field of view, and therefore to simulate their observed coma a large computational domain is required. In contrast to the simulation of the near-nucleus coma, two additional factors need to be considered when modelling structures which are tens of thousands of kilometres from the surface: (1) processes of photodissociation and (2) the unsteady character of the flow. For example, in Rodionov and Crifo (2006) an attempt was made to explain the nature of the weak spiral structures seen in ground-based observations and, more specifically, in observations of the comet C/1995 O1 Hale-Bopp. It was assumed that water is produced by surface ice sublimation and CO coming from diffusion from the interior of the nucleus. Two processes of photodissociation were taken into account: H_2_O → OH + H and OH → O + H. These two processes have characteristic rates of $\sim (1-3) \cdot 10^{-5}/r_{h}^{2}$ seconds. At a heliocentric distance of 1 AU their importance thus becomes apparent at a distance of $10^{4}~\text{km}$ from the nucleus. The rotational period of Hale-Bopp is 11.4 hours. The gas expanding from the surface travels about $2 \cdot 10^{4}~\text{km}$ during one rotation. Therefore the flow in the region of $10^{5}~\text{km}$ radius is in an unsteady state. The model demonstrated the capability to generate large-scale gas spirals of the kind observed in comet C/1995 O1 Hale-Bopp, thus demonstrating that the existence of such spirals does not require surface heterogeneity. On the other hand, this result does not in itself exclude heterogeneity either.

Including in the model the photodissociation process may be important not only for a large region of simulation. For example, in observations of comet C/1996 B2 Hyakutake, a bow structure was noticed in spectra showing the minor species (like OH) at the distance $\sim 10^{3}~\text{km}$. Even though at this distance the abundance of OH is much lower than that of H_2_O, the process of photodissociation is of crucial importance for the spatial distribution of OH.

In the case when activity is strong (usually at small heliocentric distances) and the flow is in the continuum regime in the near-nucleus region, the approach which describes the dusty gas flow as a fluid is justified. But when a comet is weakly active or has less active regions the flow in the coma can be rarefied ($Kn>0.01$). In the rarefied flow, the collision time between molecules is long compared with transport times. The mean free path between collisions is comparable to or larger than the flow gradient. In these cases the validity of the hydrodynamic approach is questionable (see e.g. Finklenburg et al. 2011). The Direct Simulation Monte Carlo (DSMC) method (Bird 1976, 1994) is based on the kinetic description of the flow and can be applied at any degree of rarefaction.

The first kinetic model of the coma based on the DSMC method was proposed in Combi (1996). This model takes into consideration gravity, intermolecular collisions, photochemistry, and radiative heating/cooling. It was applied for the simulation of an axisymmetric pure water expansion from a non-spherical nucleus. The boundary conditions were postulated in a simplistic manner – it was assumed that the nucleus has a uniform surface temperature, the gas is at rest on the surface with the density varying as the square of the cosine of the sub-solar angle. Gas emission on the night side was set to be zero. The study was carried out for the case $Kn \sim 10^{-4}$ (based on the radius of the nucleus and the mean free path in the sub-solar point). It was shown that kinetic effects become important in the periphery of the jet and on the night side in the immediate vicinity of the nucleus. At the same time, comparisons with hydrodynamic simulations of the same problem show excellent agreement in the dense regions of the flow.

A series of papers (Crifo et al. 2002b; Zakharov et al. 2003; Crifo et al. 2003; Lukyanov et al. 2005; Zakharov et al. 2008; Crifo et al. 2004) was devoted to the comparison of the fluid (the Euler and the Navier-Stokes equations) and the kinetic (DSMC) description of the coma. Several nuclei with some typical geometrical or compositional features were studied: spherically homogeneous and inhomogeneous, aspherical with a cavity fully sunlit or partially shaded. The studies were carried out in the range of $10^{-7} < Kn < 10^{-3}$ (computed for the gas parameters at the sub-solar point and the radius of the nucleus). The results showed that: (1) on the dayside both approaches agree quite well; (2) on the night side, where the local Knudsen number $Kn=\frac{\lambda |\nabla n_{g}|}{n_{g}}<0.1$, the agreement is satisfactory as well; (3) if the night side is active then the agreement is satisfactory even for the local Knudsen number $>0.1$.

A kinetic model (based on the DSMC method) of the axisymmetric multispecies (H_2_O, OH, O, H_2_, H, and CO) coma with a realistic thermophysical model of outgassing from the surface of the nucleus was presented in Tenishev et al. (2008). The surface was assumed as a porous layer of solid grains and ice particles which sublimate under solar illumination. The thermophysical model (Davidsson and Gutiérrez 2004, 2005, 2006) takes into account the processes of the gradual absorption of solar energy in the surface layer due to a finite optical opacity of the ice/dust mixture, the thermal re-radiation, the re-condensation of the gaseous constituents of the coma on the surface of the nucleus, the solid-state heat conduction, the subsurface ice sublimation and re-condensation, and the subsurface transport of mass and energy due to gas diffusion.

This gas coma model (Tenishev et al. 2008) was further completed by the dusty coma model (Tenishev et al. 2011). The numerical model of the dusty coma was constructed in the spirit of DSMC for the gas (i.e. the dust phase in the coma is represented by a large but finite number of model particles that represent real dust grains). It was assumed that the dust motion is governed by the gas drag and nucleus gravity force. The distinctive feature of this model is the self-consistent kinetic treatment of both the gas and the dust phases of the coma. Though, the numerical simulation performed for the comet 67P at heliocentric distances from 1.29 to 3.25 AU and the dust-to-gas mass production rate $\sim 1$ have shown that the back coupling of momentum exchange in the coma and the heating of the gas by collisions with the dust is negligible.

In Fougere et al. (2013) the model of Tenishev et al. (2008) and Tenishev et al. (2011) was applied for modelling the heterogeneous ice and gas coma of comet 103P/Hartley 2. Axisymmetric simulations were performed for a gas coma (H_2_O and CO_2_) using a realistic bi-lobed shaped nucleus with dimensions corresponding to the ones observed by the EPOXI mission (A’Hearn et al. 2011). The model included the possibility that icy grains can sublimate while being carried away from the nucleus and constitute an additional source of water in the coma. A model of pure icy grains ($1~\upmu \text{m} \leq \cdots\leq 8~\text{cm}$) was used with equilibrium temperature and sublimation rates which were computed using the energy balance between sublimation of volatiles, and absorption/emission of radiation (see Fougere et al. 2012). The adjustments of the model were made based on comparisons of (1) computed column densities of H_2_O, CO_2_ and observed brightness of water, and CO_2_; (2) computed and measured IR spectra of H_2_O (2.7 μm) and CO_2_ (4.3 μm). The results have shown that most of the water released by Comet Hartley 2 comes from the sublimation from icy grains as they are carried away from the nucleus by the gas drag. A strong correlation was found between the icy grain and the CO_2_ densities, which raised the hypothesis that the icy grains were lifted by the hyper-volatiles in collimated jets mostly present at the sub-solar lobe of the nucleus.

Up to the rendezvous of Rosetta with comet 67P in 2014, the coma modelling was focused mostly on possible scenarios of cometary activity and trying to study potential influences of different factors.

With Rosetta’s data obtained from close distance to the comet by different instruments during a long period of time a new stage of coma modelling has begun. The modelling of 67P can be based on much more precise data and the models are more tightly constrained. New models build on and combine previous studies of particular effects. Now the main problem is not the general simulation of the dusty gas flows but rather the derivation of the boundary conditions via fitting of simulations to the available observational data (e.g. Bieler et al. 2015a; Fougere et al. 2013, 2016b; Marschall et al. 2016, 2017; Zakharov et al. 2018a; Marschall et al. 2019; Combi et al. 2020; Marschall et al. 2020b). We discuss this link of the nucleus surface (i.e. the boundary conditions) to measurements in the next section.

## Linking Coma Data with the Surface

One of the most important goals of Rosetta was to deliver observations which can be used to piece together a consistent picture on the spatial distribution and time evolution of surface sources for gas and dust. It had been envisioned that inverting the remote sensing measurements was going to yield the much needed constraints on the “intrinsic” scales of gas/dust sources which can then be linked with specific surface features identified from the visible wavelength images. We will discuss this approach and its drawbacks in Sect. 4.1. In addition, the computational and numerical modelling has advanced to such a degree by the time Rosetta arrived at the comet that rarefied gas flow calculations in three-dimensional domains have become feasible, simultaneously coupling the modelling of measurements of different instruments. This approach we will label “forward” modelling, and by means of varying the input parameters we can fit the observed data, hence assess the physics of the comae. We will explore this approach and its drawbacks in Sect. 4.2 as well as the nature of how dust filaments (jet-like structures) can arise (Sect. 4.3). In Sect. 4.4 we will discuss the distribution of sources from the different models currently used, how they differ, and how conflicts between them can be resolved. Finally, in Sect. 4.5 we give an overview of how VIRTIS data has informed our inner comae knowledge.

### Inversion Techniques for the Inner Gas and Dust Comae

#### Inverting In-Situ Gas Measurements

On August 6, 2014, the Rosetta spacecraft had for the first time reached a cometocentric distance of about 100 km, which marked the beginning of near-nucleus operation at comet 67P. At this time the ROSINA instrument suite began to record signals consistently above (spacecraft) background (Altwegg et al. 2015). The direct in-situ ROSINA data provided estimates on the local number density of many molecular species. Each measurement was registered on the digital terrain model of 67P at the sub-spacecraft position. The surface outgassing rate may then be obtained, in the case of the simplest inversion approach, from the “Haser model” (Haser 1957) where the density measured at the spacecraft is simply nadir-projected onto the nucleus shape being scaled using the “$r^{2}$-law” of Eq. (4). It was rather surprising to find this approach to give reasonable results regarding the diurnal evolution of outgassing (Bieler et al. 2015b). Given the large field of view (FOV) of ROSINA and the complex nucleus shape and detailed pattern of illumination it was expected that this kind of nadir projection would not be able to yield a reasonable model of gas coma evolution. In addition the model assumes a collisionless flow, an assumption that can be safely considered only for outgassing conditions at large heliocentric distances $>3.0$ AU (in the context of 67P). Applying this model to measurements in a given time period it has been also used to estimate the global gas production. The validity of this approach is limited by its assumptions, and accurate global production rates were also not expected from this method. In addition, locations of outgassing on the surface cannot be obtained from this simple inversion approach. This has been shown in Marschall et al. (2017) where emission from cliffs and the Hapi region resulted in equally good fits of ROSINA data as with inhomogeneous outgassing involving the entire surface. In short, the results from a simple inversion must be considered with some care.

A more sophisticated inversion approach was applied to constrain the gas sources at the surface from the ROSINA data in Kramer et al. (2017) and Läuter et al. (2019). In their work they assume gas sources on a shape model with roughly $4'000$ facets that emit gas with a certain opening angle with respect to the local surface normal. The gas flow in this model is still assumed to remain collisionless in the entire computation domain, hence, each measurement of ROSINA is simply the (weighted) linear combination of the contribution of all these facets. A global minimisation method of reducing the difference between thousands of measured and simulated ROSINA densities yields the surface facet weights. The facets with the largest weights are the “local” surface sources of gas. The advantage of this approach is its speed, hence it can be applied to data for the entire orbit. The downside of the method is that, even if it yields a good estimate of the global production rate, the resulting source distribution should be treated with care because it is not the outcome of a model that takes into account the necessary physics. For one, the basic assumption that each surface facet contributes linearly is not justified because the gas molecules originating from different areas do interact with each other thus altering the flow. Other challenges of inverse models will be presented in Sect. 4.4.

#### Inverting Remote Sensing Measurements

At the onset of near-nucleus observations (Aug. 2014) the microwave instrument for the Rosetta orbiter, MIRO, was already monitoring the water production rate for more than two months (Biver et al. 2015), triggered by the first detection of water activity on June 7, 2014 (heliocentric distance of 3.925 AU) of merely 300 g/s (Gulkis et al. 2015). In addition of retrieving molecular densities along the line-of-sight (LOS), the line shapes in the MIRO measurements carry further information on gas velocity (projected on the LOS) as well as kinetic temperature of the gas. The measured photons in the sub-millimetre wavelength range originate from the rotational transitions of molecules which are spaced closely in energy and thus still efficiently thermalised in the inner coma due to inter-molecular collisions. Nevertheless, in the context of cometary comae the population of rotational levels does deviate from local thermodynamical equilibrium (LTE) at some distance from the nucleus (Zakharov et al. 2007; Davidsson et al. 2010), and forward models of MIRO spectra have to generally take this into account (Lee et al. 2011). The velocity profile along the LOS can be estimated from the Doppler shift of the frequency of the measured line shape of a particular rotational transition, as will be discussed later. In this respect, the MIRO observations are truly exceptional in providing far more information constraining the coma’s dynamical state than, for instance, the point measurements of density by the ROSINA instrument suite. However, the process of extracting this entangled information from the spectra requires more sophisticated analysis and coma models; this is still a topic of active research.

The first parametric inversion estimating simultaneously the water production rate and terminal velocity along the LOS was carried out by Lee et al. (2015) on a subset of MIRO data taken in nadir geometry in August, 2014. During this period the comet was only weakly active (Q[H_2_O] usually below $10^{26}~\mbox{s}^{-1}$). These conditions in fact provided two advantages for the interpretation of data and played a role in the selection of a particular parametric coma model. First, the main isotopic line $\mbox{H}_{2}^{16}\mbox{O}$ at 557 GHz was still not so optically thick to lead to saturated line cores, such that the line area ($\mbox{K}\,\mbox{km}\,\mbox{s}^{-1}$) remains quasi-linearly dependent on the water column density along the LOS. Second, due to the relatively low optical thickness the determination of expansion velocity is somewhat easier as the line cores are not saturated. The coma model in the cited work was compressed to five parameters (Q, a, b, $\mbox{v}_{0}$, c), where Q is the water production rate, (a, b) are related to the power law approximation of the temperature profile required to simulate the MIRO spectra, and ($\mbox{v}_{0},\mbox{c}$) are used in a tanh function used to model the velocity along the LOS. The solution of the inverse problem is performed within the framework of optimal estimation (Rodgers 2000) which provides a statistically sound regularisation and error characterisation of the results given that the required assumption can be fulfilled. As acknowledged in the work of Lee et al. (2015) an important but often neglected consideration for non-linear inverse problem solution concerns the degree of uniqueness. Generally, it is not a simple matter to always demonstrate that a global optimum has been found for each processed MIRO measurement. The work is usually tedious and typically relies on a Monte Carlo type sensitivity analysis.

**Fig. 7:**
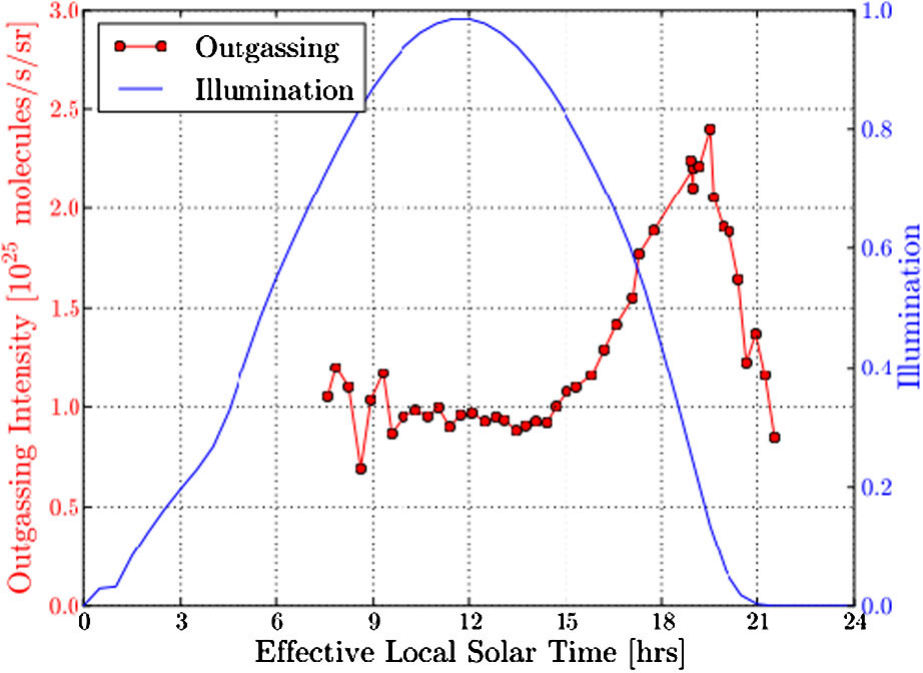
Local water production rate versus effective local solar time. The effective local solar time is determined by calculating the angle between the direction of the local normal direction at the location of the MIRO centre beam and the Sun direction. Adapted from Fig. 13 (Lee et al. 2015)

**Fig. 8:**
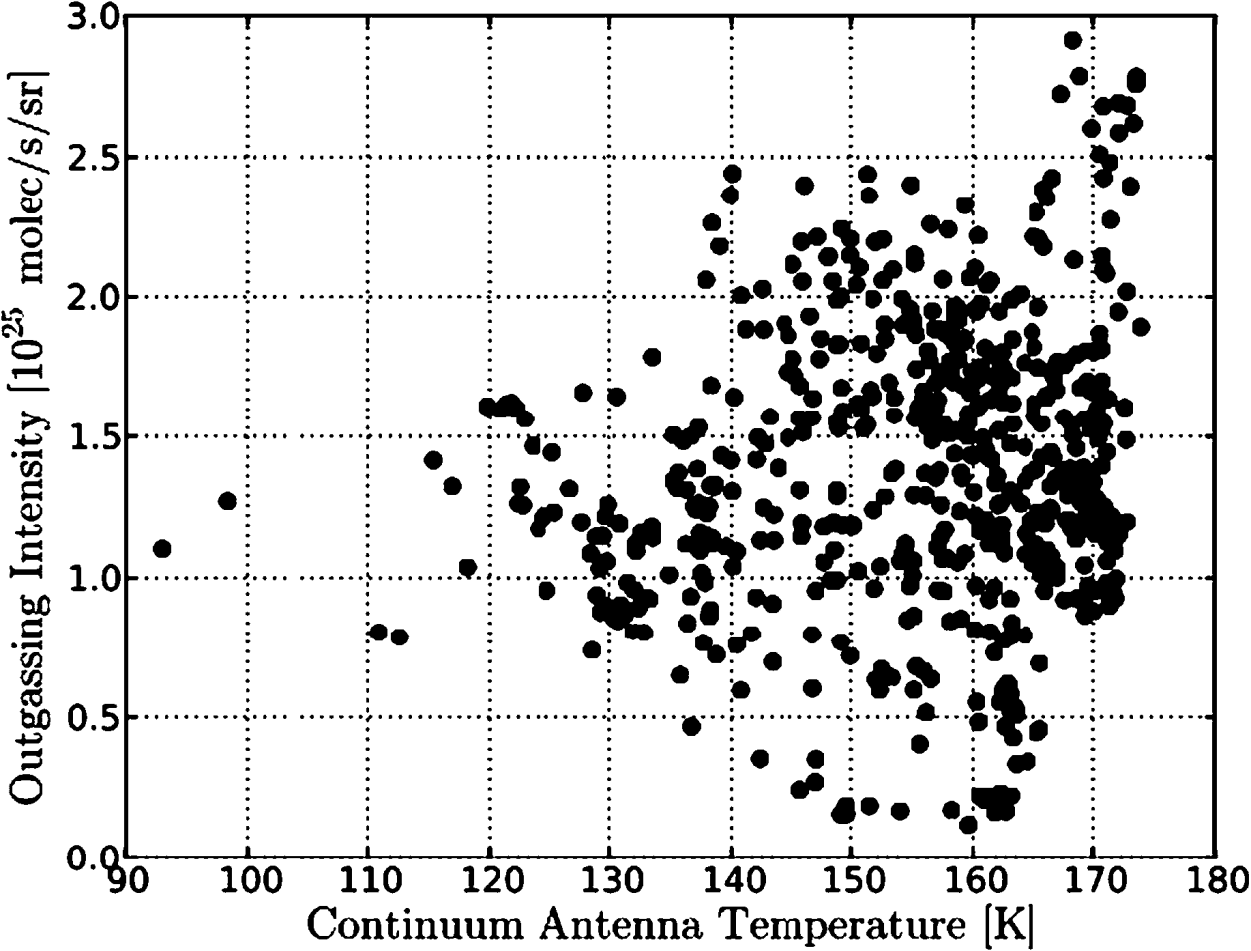
Adopted from Fig. 11 in Lee et al. (2015) which shows the distribution of the water outgassing intensity as a function of MIRO sub-millimetre antenna temperature registered at the MIRO beam location on the nucleus. No obvious correlations can be observed

These first MIRO inversions were in an apparent conflict. It was recognised since the earliest observations that the water outgassing originates from the illuminated hemisphere, with the largest water density found when pointing toward the “neck” region of 67P. This was an unambiguous conclusion even with 1D Haser models used in the MIRO measurement reduction. However, when individual MIRO observations were projected onto the surface (at the beam-nucleus intersection location) and correlated with local time (Fig. 7) the expected relationship for column density correlation with mean illumination (or local time) was not found (Lee et al. 2015). Similarly, no obvious correlation was discovered between water flux and the surface continuum temperatures (see Fig. 8). It was speculated that this is a result of heterogeneous subsurface ice distribution, such that the largest contribution of molecules into the MIRO beam comes from other regions than the location of the FOV.

**Fig. 9:**
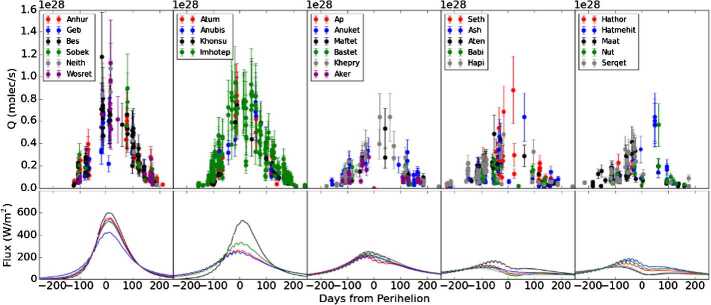
(Top panels) Water production rate 250 days before and after perihelion sorted by regions; adopted from Fig. 6 in Marshall et al. (2017). In particular we highlight the northern region results in the fourth panel of the figure where five northern regions are plotted: Seth, Ash, Aten, Babi, and Hapi. These regions receive relatively low mean daily solar flux (bottom panels), however, Seth and Ash are still quite active as determined by MIRO

A different approach to the inversion was taken in the work of Marshall et al. (2017), relying on constructed look-up tables for the line areas of the MIRO main spectroscopic transitions $\mbox{H}_{2}^{16}\mbox{O}$ and $\mbox{H}_{2}^{18}\mbox{O}$. In addition, due to the simplicity of modelling the nadir geometry, the limb measurements were not considered in this work. In order to be able to deal with the different optical thicknesses in these transitions a line area ratio was formed and used to invert individual MIRO measurements for water density. As in previous works, all observations were also registered to the surface at the location of the MIRO beam. The look-up table method’s main advantage is computational speed, and nearly the entire MIRO data set has been processed such that water production rate can be statistically examined as function of time and space. It is also worth pointing out that, just as in Lee et al. (2015), the water production rate was calculated assuming a 2 km sphere with Haser-like water outgassing, that is, each observation was treated as if a spherical nucleus was observed. It was referred to as “local effective Haser” production rate by Marshall et al. (2017). With the processing of the entire data set available at the time several interesting findings were made. First, the plot of production rates as functions of heliocentric distance showed a delayed peak (20–40 days) with respect to perihelion. This feature was also observed with ROSINA data (Hansen et al. 2016; Läuter et al. 2019; Biver et al. 2019), but it is not yet clearly explained by theoretical thermal models. Second, the calculated slope of the heliocentric water production rate averaged as a function of latitude can be very well described by a purely illumination driven model assuming surface ice activity from each facet of the digital shape model. Third, a relatively high production rate (around perihelion) is implied by the MIRO data in the northern regions of the comet (especially the Seth region), which according to the shape model experienced a rather low illumination (mean daily flux of $50~\mbox{Wm}^{-2}$), see panel 4 in Fig. 9. Incidentally, the Seth region is adjacent to the Hapi region, both located in the northern hemisphere neck. Despite the successful application of this approach a question remains: Why does the “local effective Haser” coma model work so reasonably well explaining the measurements, although we see such a morphological heterogeneity on the nucleus? And, how accurately can we recover the 3D spatial pattern of activity from 1D models of column density? These issues will be discussed later.

**Fig. 10:**
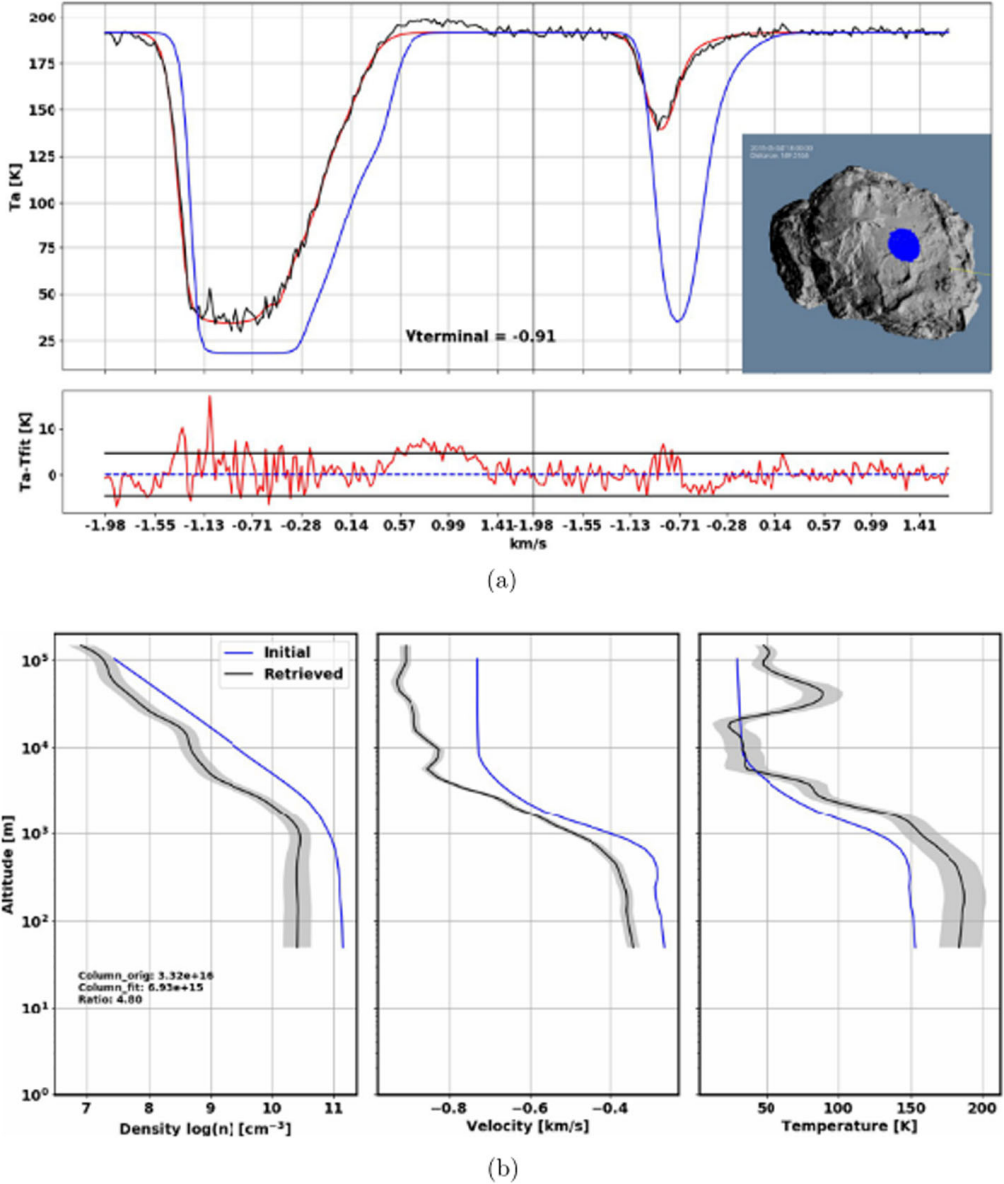
The top panel shows three spectra for the 557 GHz transition of $\mbox{H}_{2}^{16}\mbox{O}$ (stronger absorption) and 548 GHz $\mbox{H}_{2}^{18}\mbox{O}$ (weaker absorption) for the case of a nadir viewing geometry (footprint illustrated in inset image). The black line shows a MIRO measurement, red line represents simulated spectra after final retrieval iterations and the blue curve is a synthetic spectra for the a-priori profile supplied by the 3D model. The middle panel depicts the residual between measurement and the best fit (red line). The bottom three-panel plot shows vertical profiles of (left) number density, (middle) expansion velocity, and (right) kinetic temperature for initial conditions as blue lines, and final retrieved profiles as black lines. The shaded region represents a 2$\sigma $ component of uncertainty due to measurement random error propagation. Figure adopted from Fig. 12 in Marschall et al. (2019)

A yet different approach to inverting the nadir MIRO measurements taken around the equinox conditions in May 2015 is described in the multi-instrument study of coma data in Marschall et al. (2019). In the present section we treat only the noteworthy details of the inverse problem related to the MIRO spectra, while the general 3D DSMC modelling and comparisons to multi-instrument data are further discussed in Sect. 4.2 in this chapter. In the cited paper, the simultaneous inversion of the two water transitions ($\mbox{H}_{2}^{16}\mbox{O}$ and $\mbox{H}_{2}^{18}\mbox{O}$) was performed in a framework very similar to the one of optimal estimation formalism as used in Lee et al. (2015). However, the vertical profiles of density, temperature, and velocity along the LOS were discretised along the radial direction (along the LOS). The physical and stabilising factor of this non-linear inversion was to start the retrieval from self-consistent a-priori profiles and co-variance matrices constructed from the output of a 3D DSMC model. The retrieval iteration was allowed to rigorously fit the measured signal, which revealed several never before reported features that remain a challenge to explain with the current understanding of gas flow physics near the nucleus. An alternative explanation is that discrepancies in the results are due to some missing physical processes driving the rotational populations of water molecules not taken into account in the current radiative transfer models. The first such surprise was an ubiquitous presence of a “red” emission wing in the nadir absorption spectra at the 557 GHz transition (see e.g. Fig. 10). The MIRO observations record enhanced radiance above the continuum temperatures for red-shifted frequencies of the line, implying a stagnant (not expanding) or tangential flow to the MIRO’s LOS of the near-nucleus gas field which is also significantly warmer (by 10–20 K or more) than the surface (the near sub-surface in the case of sub-mm wavelengths). The second unexpected finding was a temperature enhancement for several studied cases in the altitude region of 30–80 km. There were several speculations offered how to explain this feature, but rigorous physical modelling is yet to be constructed. Currently there is no indication that this feature is an artefact of the data calibration. In addition, the retrieved velocity profiles show evidence just above the 2-$\sigma $ uncertainty levels that acceleration continues up to an altitude of 20–40 km, and the radial profiles where the transition into free-molecular flow is expected are not necessarily smooth. Finally, the retrieved water column densities are found at least a factor 2 smaller than the modelled ones, which also seems to be a robust result. Figure 10 illustrates these features.

**Fig. 11:**
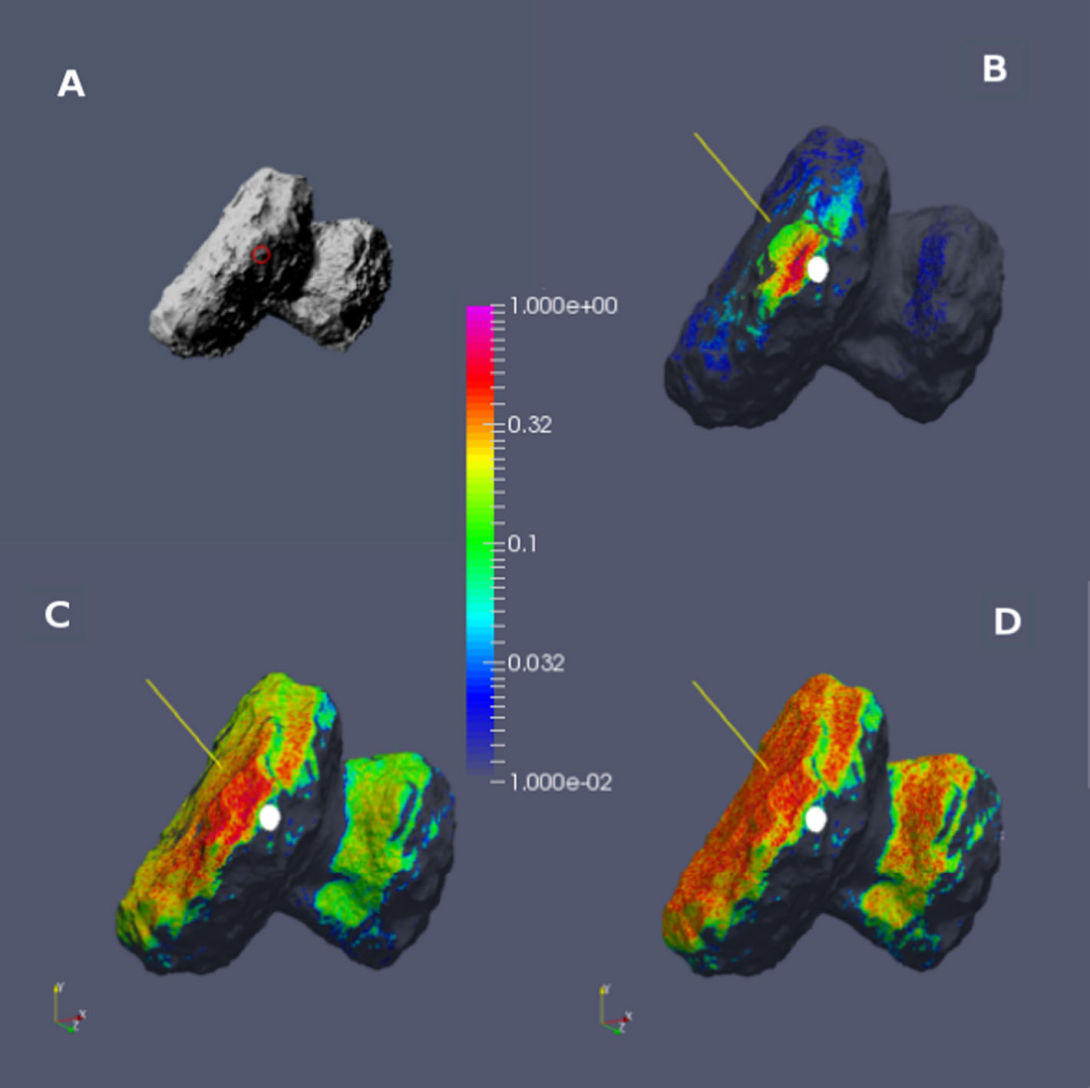
Panel (A) shows illumination conditions for a given case of MIRO nadir viewing geometry, with beam footprint located (red circle) at the terminator region on the larger lobe of 67P. Panels B, C, D show horizontal distributions of H_2_O density at all the different facets visible from a grid point at 2.5 km (panel B), 5 km (panel C), and 20 km (Panel D). The grid points are located inside the MIRO FOV, and the camera position in this image coincides with the S/C centred on the MIRO beam centre. The colour bar is in relative units (with respect to the facet of maximum contribution to a given grid point). This figure illustrates that even at heights of 5 km and especially 20 km the sample point on the LOS may receive contribution from a large area on the surface. This figure is adopted from Fig. 6 in Rezac et al. (2019)

All nadir remote sensing observations have a limit on the vertical resolution they can achieve. That is dictated by the physics of the line formation and its dependence on the parameters we want to retrieve. In addition, the vertical smoothing also varies for the different observing geometries and the instrument’s FOV. The task of inverting measurements to constrain local sources of water activity on the comet surface turns out to be a more complex problem than initially thought as recently reported in Rezac et al. (2019). That study relied on a simplified 3D approach to evaluate the relative water density contribution from facets inside the MIRO beam versus all other visible (and active) facets to a given grid point along the LOS of MIRO. Under the assumption of homogeneous, illumination driven outgassing, they found that less than 1% of the water column density originates from facets directly in the MIRO FOV. This implies that there is little hope that individual inversion can discriminate active from non-active regions at the scale of several FOVs (see Fig. 11). Additionally, it also confirms, or explains, why the beam-averaged illumination, local time or continuum temperatures may not correlate with the observed water density as reported in Lee et al. (2015). However, this result does not support the original speculations that this weak correlation necessarily implies a heterogeneous surface activity. To confirm this a more sophisticated 3D spatial inversion is recommended. And finally, it also provides a qualitative argument why the 1D Haser coma model when applied to MIRO, but also to other instruments, achieves reasonable success in explaining the measurements to first order. It appears that the same physical limitations as to why observations cannot provide a unique solution for the local sources of activity are also the reason why the Haser density distribution is a reasonable approximation for a first rough approach. A similar conclusion on the degeneracy of localising surface sources of gas activity was already drawn in Marschall et al. (2017) in application to ROSINA/COPS data.

#### Inverting Dust Measurements

The processes of interaction between coma and nucleus surface have been debated since ground based observations revealed anisotropies in cometary comae. It is well known that active comets typically display regions of higher gas and dust density in their coma, often named “coma structures” by astronomers, or sometimes “jet-like” features to highlight their apparent collimated nature. We focus here on the large-scale phenomena which span the full coma and refer the reader to Vincent et al. (2019) in this book for a review of resolved jet-like features and their relation to the surface topography.

While there is no question about the presence of coma structures, extending for tens of thousands of kilometres away from the nucleus, their nature has proven more elusive. Two prominent questions must be answered: Is the collimation real? If yes, are coma structures a signature of compositional heterogeneities on the nucleus surface, or simply due to the topography?

In order to relate nucleus and coma structures, one must first determine whether the “jet-like” appearance actually implies a narrow collimation or if is just an illusion due to the geometry of our observations. It was recognised early by Sekanina (1981, 1987) that a dust/gas source at the surface of a rotating nucleus will automatically lead to a fan-shaped structure in remote observations. Indeed, as the nucleus spins the flow structure emitted from an active source will sweep the surface of a cone, centred on the apparent source and aligned with the spin axis of the nucleus. Because of the scale and resolution of ground based data, this cone cannot be observed fully but only its edges. This is because we are looking at the two dimensional projection of a tri-dimensional structure. As such, the density of gas/dust per pixel appears much higher on surfaces parallel to our line of sight, than on surfaces parallel to the image plane. Hence, the collimation and fan-like appearance of coma structures is more a result of the nucleus rotation than of an inherent mechanism leading to the release of cometary material in a spiral pattern.

This hypothesis has been put to test by the long-term monitoring of many comets. Over the course of months they develop various coma structures which can be used to constrain the nucleus spin axis. This technique has proven to be quite reliable; several authors used coma structures to determine the rotational state of comets 9P (Vincent et al. 2010), 103P (Lin et al. 2012), or 67P (Vincent et al. 2013) with excellent agreement between predictions from ground-based data and in-situ observations by space missions.

One can even go one step further to constrain the activity. Provided monitoring over several comet rotations, it is possible to determine whether the source from where the fan appears to originate is constantly active or only during the local day. This puts constraints on the type of sublimation driving the activity. Such considerations were used for instance to derive the location of a CO_2_ source on comet 9P (Vincent et al. 2010) and seasonal variations of activity on comet 67P (Vincent et al. 2013).

Additional considerations such as the curvature of coma structures from radiation pressure are also used to retrieve information on the dust present in the coma, for instance grain size and velocity.

These observations tell us that activity appears to be more prominent in certain areas of the nucleus and that the distribution of active regions varies with seasons, typically following the subsolar latitude. Yet, they do not address the nature of those sources. In fact, the inversion of ground based data shows that flows of gas and dust appear to originate from discrete areas but this can be equally explained by real “active regions” (intrinsically different from surrounding areas) or by topographic collimation (large areas are active, but local height variations confine the flows, giving the illusion of a discrete source).

Spacecraft visiting comets have observed jet-like features at high spatial resolution and for many nucleus rotations. They revealed that both explanations are valid and play a role on comets. Regardless of the distribution of ices, local topography will always lead to heterogeneities in the gas flow (e.g Crifo et al. 2002c; Shi et al. 2018). At the same time, it is possible to find areas of the nucleus that are actually releasing more material, without a focusing effect induced by topography. Long-term monitoring at close distances also confirms that the distribution of discrete sources follows the solar insolation, with maximum density of local sources close to the subsolar latitude (Lin et al. 2015; Vincent et al. 2016c; Schmitt et al. 2017; Lai et al. 2016). In the case of 67P these inversions relied on the OSIRIS camera on-board Rosetta that detected the light reflected from the dust coma and jet-like structures within it. If the same dust filament is observed in at least two images a geometric inversion of the jet-like feature can be performed. Each image defines a plane within which the jet-like feature lies, and the intersection of the two planes of the two images gives a single line (except if the planes are parallel), the intersection of which with the surface can be determined, and thus the footprint of the feature on the surface can be found. This has been done by Vincent et al. (2016c) and Lai et al. (2017). They have found that the footprints correlate heavily with cliffs on the comets. Increased activity near freshly eroded cliffs and scarps was also found by Fornasier et al. (2017). The only caveat we would like to point out here is that the method assumes that the dust jets are not curved and are traced straight down to the surface. The actual sources of the jet-like features are mostly not actually seen because, as we mentioned in the introduction, the brightness of the dust is much lower than that of the illuminated surface at least for 67P. So, any curvature of these features close to the surface adds to the uncertainty on the origin of the dust feature. Furthermore the trace-back of these dust features does not yet imply that there is a distinct source at the surface. We will discuss this in more detail in Sect. 4.3.

Finally, the GIADA instrument on Rosetta has measured the momentum and size of dust particles for comet 67P, and thus it can be attempted to trace-back these particles to the surface. Because their speeds are rather low ($\sim \mbox{m/s}$) the trace-back problem gets challenging, and therefore the uncertainties on the origin of the dust particles on the surface is very large. This has been shown in detail by Ivanovski et al. (2018). Thus, GIADA does not yield the locations of the dust particles’ origins to a high resolution, but the particles can rather only be traced to large regions of the comet (Longobardo et al. 2019).

### Forward Modelling for Constraining Emission Region

A complimentary approach to the formulation of an explicit inversion is to utilise a self-consistent 3D physics model (or 4D including the time dimension) of the coma coupled with synthetic measurement algorithms for various instruments. Under the assumption that at each narrow time interval we have a unique snapshot of the cometary state such that we can vary the input parameters in order to fit self-consistently all the measurements at the same time accounting properly for gas and dust density, temperature and velocity. We refer to this approach as “forward modelling” (or “forward fitting”). From a strictly mathematical point of view, this approach can be regarded as an incomplete optimisation (inversion) problem, where only a single (or few) iteration(s) are performed, but a convergence is not reached.

The advantage is to engage complimentary information from different instruments on the state of the coma (gas/dust density, temperature, velocity) to guide the full 3D comae physics model. For completeness, and perhaps for stimulation of future work, we note that there is an entire research field devoted to such problems in application to weather forecasting. There, vast real-time data sets of measurements are assimilated into global weather models through so-called 4D-variational assimilation procedures based on the Ensemble Kalman Filtering method (Rabier 1997; Rabier et al. 2000). In this section we will describe efforts applied only to the Rosetta data in terms of forward fitting using 3D DSMC models.

**Fig. 12:**
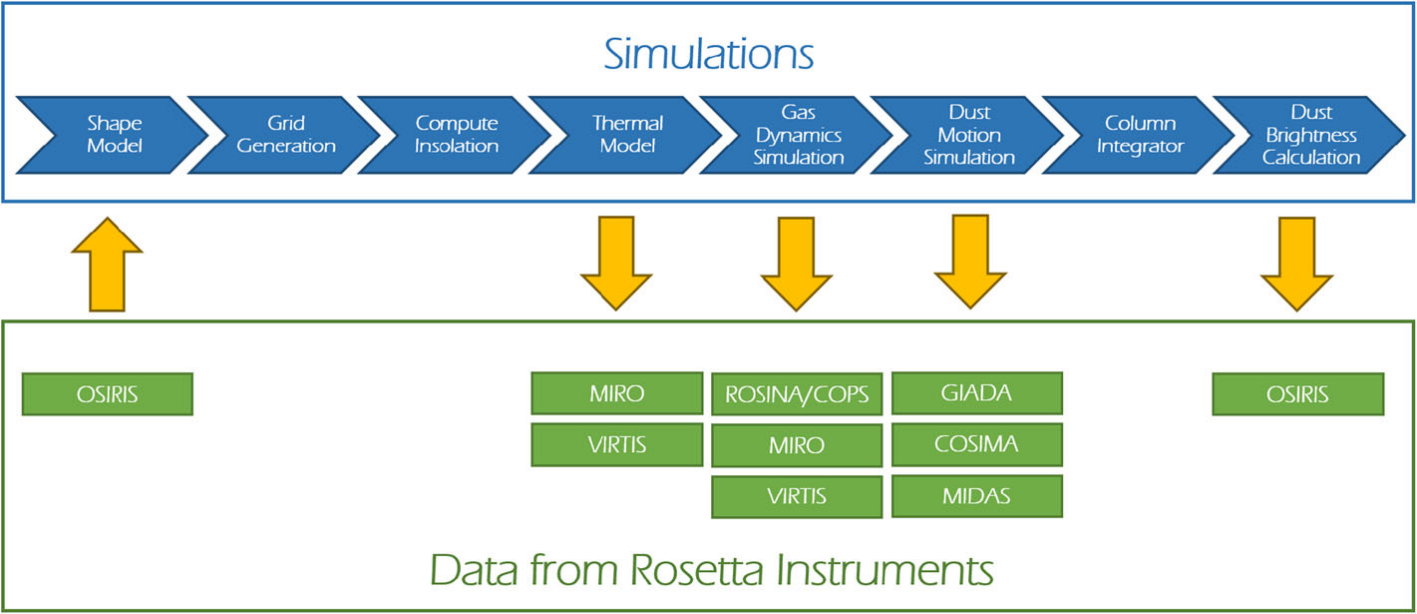
Sketch of the simulation sequence representative for the forward models and how the different pieces can be used to compare with the data (adapted from Marschall et al. 2019)

This, what can be considered a common approach, has been applied by several research groups and the results published in many papers (Bieler et al. 2015b,a; Marschall et al. 2016; Fougere et al. 2016b,c; Marschall et al. 2017; Zakharov et al. 2018a). The basic simulation sequence – which is quite similar for all groups – is illustrated in Fig. 12. The different groups use very similar approaches to fit mostly ROSINA data of the local gas density either using a fluid or DSMC approach for modelling the gas dynamics. All groups start with a nucleus shape model upon which the real illumination conditions are calculated taking into account shadow casting. This is used as an input to a thermal model that determines the gas production rates and temperatures at the surface which are the inputs for the gas dynamics programs, the outputs of which can be compared to measurements of the gas instruments ROSINA, VIRTIS, and MIRO. The gas flow field can then be used to simulate the dust flow which can either be compared to results from the in-situ dust experiments GIADA, COSIMA, and MIDAS, or, by means of column integration and scattering theory, to OSIRIS dust coma data. The strong advantage of this approach is that it provides a physical connection between the emission of gas at the surface and measurements in the coma. These models should be favoured over others which run fast but don’t include the necessary physical processes. The major drawback of this approach is that it is very computationally expensive and thus very time consuming. This in turn makes a systematic examination of the large parameter space challenging.

Although the basic approach of all groups using these forward models is basically the same they have different optimisation schemes. The group from Michigan, US, (e.g. Fougere et al. 2016b) have optimised the sources of gas at the surface using spherical harmonics. The group in Bern, Switzerland, (e.g. Marschall et al. 2016) assume that the emission strength correlates with morphological regions and optimise the fit according to those regions. Finally, the group in Paris, France, (Zakharov et al. 2018a) run an iterative model where an initial DSMC simulation is run and then the measurements are compared to the model. The deviations of the model to the data adjusts the initial conditions of the source distributions at the surface, by tracing the measurements to the surface within the flow field and changing the respective weights. A new DSMC simulation is run and the iteration continues. We will discuss the similarities and differences of the results of the different groups in Sect. 4.4.

### Mechanisms that Result in Filament Structures

In the previous section we have discussed the forward modelling approach for the gas coma. Here we want to summarise some results from forward modelling of the dust coma. The simplest case to produce a dust jet-like structure is having a surface source, i.e. a strong enhancement of the emission in a confined area in contrast to its surrounding. Such a source can occur due to compositional heterogeneities of the nucleus itself or evolutionary differences in surface properties (e.g. thickness of isolating dust layer). But this is not the only way to produce features in the coma, and the interpretation of these features should thus occur with great caution. Crifo et al. (2002a) have pointed out that dust structures in the coma don’t require sources on the surface. Rather a non-spherical shape is sufficient to dynamically produce such features by dynamical means. The surface topography focuses gas and thus also dust flows resulting in higher density regions within the coma. A similar case has been pointed out by Shi et al. (2018) where the sublimation along the morning terminator line can produce pronounced features because the terminator is not a straight line. More complex examples of how these dust structures form are illustrated in e.g. Marschall et al. (2017). In addition the viewing geometry plays an important role. Because imaging instruments observe column-integrated quantities, something that might appear to be a fan like structure in one orientation will look like a very fine line in another. Furthermore, variations of the surface illumination due to topography (e.g. due to shadow casting) can produce heterogeneous emission patterns leading to jet-like features that are not linked to any compositional heterogeneity of the nucleus. Jet-like structures in the dust coma can thus arise due to a wealth of reasons. These structures can occur due to genuine surface sources, dynamical focusing caused by topography, emission variations caused by topography-induced illumination conditions, and observational effects caused by the viewing geometry.

### Similarities and Differences of Models of 67P

**Fig. 13:**
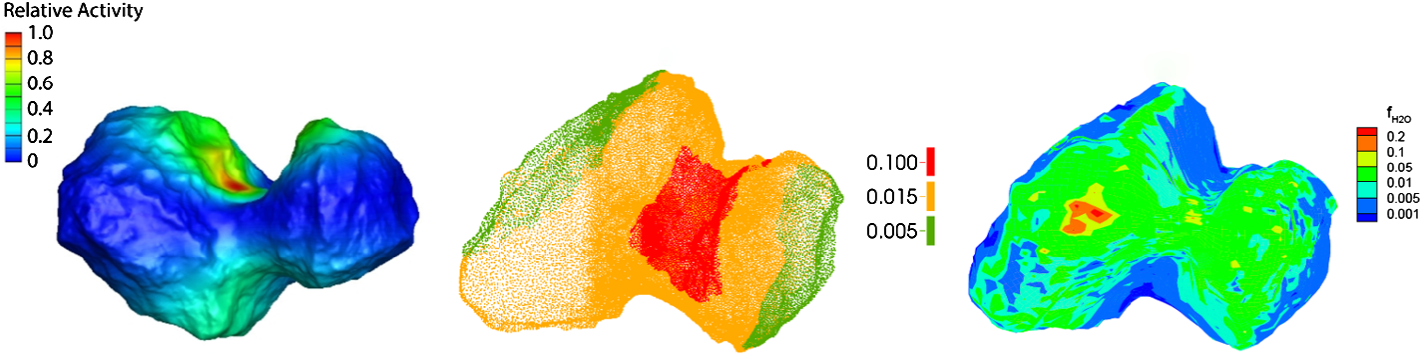
These three views of the cometary surface show the relative potential for outgassing all for northern summer, i.e. $\sim 3~\mbox{AU}$ according to three research groups (from left to right): Fougere et al. (2016c), Marschall et al. (2016), Zakharov et al. (2018a). While the two right maps show the nucleus in the same orientation the first is shown from the side

Having discussed the different approaches we can now examine the results of the different models. We will discuss only the maps of sources resulting from the physical models discussed in Sect. 4.2. One important conclusion all research groups arrive at is that the gas – and for that matter also the dust – activity cannot simply be explained by the variations in illumination as the comet rotates and the associated changes in energy input to the surface (Fougere et al. 2016b; Marschall et al. 2016; Zakharov et al. 2018a). The nucleus can therefore not be treated as a homogeneous piece of material. Given the diverse morphology of the surface this might not be too surprising but it is an important result. This does not yet imply though that the bulk nucleus is heterogeneous. Given the strong indications of mass transport and dust deposition, variations in the activity potential of the surface are likely evolutionary rather than compositionally. The strong seasonal differences are therefore mainly the result of the mass transport and not the inherent composition of the nucleus. Similar conclusions can be drawn for the mass transport observed at Hartley 2. The water source distribution derived by the different research groups is shown in Fig. 13. Two things need to be noted. The models don’t agree on the inhomogeneity. The models from Michigan and Bern agree qualitatively in that the main source of water emission comes from the north polar region (north pole in Fougere et al. 2016c and Hapi region in Marschall et al. 2016). In contrast to that the Paris group sees enhancements of the emission towards the northern parts of the head and body lobes rather than the neck region (Zakharov et al. 2018a). At this point all groups claim good agreement with the data. This seems like a contradiction but in fact it is not. On the contrary, it shows that the gas flow smears out any inhomogeneities from the surface rather efficiently. This degrades the resolution and thus the possibility of an instrument like ROSINA, measuring the gas density tens of kilometres from the surface, to accurately determine the sources. This also highlights the issues of inverse models as discussed in Sect. 4.1. The physics involved in the expansion of gas allows for multiple surface distributions to result in the same measurements. The solutions are thus naturally degenerate. But where does this leave us in our pursuit of determining the source distribution at the surface? There is a way out because the above argument only holds if we include just ROSINA data. This implies that additional data from other instruments such as VIRTIS and MIRO must be included to break this degeneracy (Marschall et al. 2019). That the solutions of the gas emission distribution are fundamentally ill-constrained below a spatial scale of a few hundred metres was shown in Marschall et al. (2020a).

The Michigan and Paris groups have also provided results for CO_2_ measurements. Both saw an anti-correlation between the distributions of water and carbon dioxide (Fougere et al. 2016c; Zakharov et al. 2018a; Combi et al. 2020). While water is concentrated to the northern hemisphere carbon dioxide is emitted predominantly from the equatorial region and southern hemisphere. The fact that there seems to be a strong seasonal effect in the emission of water vs. carbon dioxide has already been pointed out by Hässig et al. (2015) who saw strong variations in the species’ ratio. In particular they have found that the southern Imhotep region has a high carbon dioxide over water ratio during northern summer.

Finally, linking gas and dust models as done e.g. by Marschall et al. (2020b) for the entire Rosetta mission fitting ROSINA and OSIRIS data allows for a comprehensive look at the dust and gas mass loss. They found that a power-law of $q=3.7^{+0.57}_{-0.078}$ for the dust size distribution is consistent with the data and in line with independent measurements previously reported by COSIMA (Merouane et al. 2017) and GIADA (Fulle et al. 2016). This resulted in a total (integrated over one orbital period) $5.1^{+6.0}_{-4.9}\cdot 10^{9}~\text{kg}$ of dust being ejected from the nucleus surface, of which $4.4^{+4.9}_{-4.2}\cdot 10^{9}~\text{kg}$ escaped to space and $6.8^{+11}_{-6.8}\cdot 10^{8}~\text{kg}$ (or an equivalent of $14^{+22}_{-14}~\text{cm}$ over the smooth regions) were re-deposited on the surface. In turn, this led to a dust-to-gas ratio of $0.73^{+1.3}_{-0.70}$ for the escaping material and $0.84^{+1.6}_{-0.81}$ for the ejected material. This is in line with other in-situ and remote sensing estimates (Choukroun et al. 2020) but lower than e.g. Fulle et al. (2019). For a detailed discussion of the dust-to-gas ratio we refer to Choukroun et al. (2020), a dedicated review of the topic.

### Rosetta-VIRTIS Perspective on the Gas and Dust Comae

Thanks to the Visible and InfraRed Thermal Imaging Spectrometer (VIRTIS) (Coradini et al. 2007) onboard Rosetta it was possible to identify several molecule species outgassing from comet 67P starting with the first operational mission phase, when the spacecraft approached the comet at about 3 AU (Bockelée-Morvan et al. 2015). The first observed molecules forming its coma were H_2_O and CO_2_ (Bockelée-Morvan et al. 2015; Migliorini et al. 2016; Fink et al. 2016). Fluorescence due the $\nu_{3}$ band of H_2_O and CO_2_ centred at 2.67 μm and 4.27 μm, respectively, was identified. H_2_O emissions were found to be more localised in the comet’s neck, where strong dust activity was also reported as well as an enrichment of the surface ice as testified by spectrophotometric properties (De Sanctis et al. 2015; Fornasier et al. 2015; Filacchione et al. 2016b). CO_2_ emissions were more intense from the southern latitudes (Bockelée-Morvan et al. 2016; Migliorini et al. 2016). During pre-perihelion, CO_2_/H_2_O ratios of 1–4% were determined above the illuminated regions (Aten, Babi, Seth and Hapi) in the Northern hemisphere (Bockelée-Morvan et al. 2015; Migliorini et al. 2016), with column densities in the order of $(2\mbox{--}5)\cdot 10^{20}$ and $1\cdot 10^{19}~\mbox{molecules/m}^{2}$ for H_2_O and CO_2_, respectively. In August 2015, few days after perihelion, an increase of CO_2_/H_2_O was reported to as high as 30–40% (Bockelée-Morvan et al. 2016). This different distribution is a consequence of the volatile characteristics: water is less volatile than CO_2_ and hence can be activated by the direct solar illumination, whereas CO_2_ can sublimate also from regions with a lower insolation. CO_2_ emission was observed especially from the southern regions, e.g. Imhotep, that were experiencing a lower solar illumination at that observing time with Rosetta (April 2015) due to the season. On the other hand, a CO_2_ ice rich region 80 m by 60 m wide has been identified on the Southern hemisphere (especially in the Anhur region) at the end of March 2015. The same spot, observed one month later, did not show any CO_2_ ice on the surface (Filacchione et al. 2016b). Other gaseous species, including ${}^{13}\mbox{CO}_{2}$, $\mbox{CH}_{4}$ and OCS, were also identified with VIRTIS (Bockelée-Morvan et al. 2016). Their abundances close to perihelion showed that the southern latitudes were more productive in CO_2_ and OCS relative to water while a lower enhancement of $\mbox{CH}_{4}$ with respect to H_2_O was observed, due to the different illumination conditions of the two hemispheres during the different seasons (Bockelée-Morvan et al. 2016). However, the first close approach to the comet showed a CO_2_/H_2_O ratio in the northern latitudes which is believed not to be representative of the primordial values, but rather a witness of the devolatilisation process occurring in the uppermost surface layers during the several approaches of the comet towards the inner solar system. The CO/H_2_O production rate ratio shows an increase during few months close to perihelion and at perihelion as seen with Rosetta/MIRO (Biver et al. 2019), in agreement also with previous measurements (i.e. Bockelée-Morvan et al. 2016; Läuter et al. 2019). Variability with respect to sub-spacecraft latitude is also reported (Biver et al. 2019). Similarly, the CO/H_2_O ratio obtained for the southern hemisphere determined close to perihelion is compatible with that of the Jupiter Family Comets (Bockelée-Morvan et al. 2016).

The imaging spectrometer VIRTIS-M, that is, the mapping channel of the VIRTIS instrument aboard Rosetta, allowed observing the full visible and infrared spectrum, from 0.22 to 5.1 μm, acquired almost simultaneously for each pixel in an image. This technique has proven to be very powerful to identify visually on the images and also spectroscopically the emissions due to H_2_O and CO_2_ (Migliorini et al. 2016; Fink et al. 2016; Rinaldi et al. 2016). Spectral continuum radiance, primarily attributed to scattering from dust, can be modelled with a fourth-degree polynomial in the 2.5–3.0 μm range, around the $\nu _{3}$ fundamental H_2_O band, and with a second-degree polynomial for the CO_2_ band (4.0–4.4 μm range), and can be removed to calculate the gas column densities. Although it is not possible to directly infer the exact spot on the surface where the observed gas emissions originated from, due to difficulties in identifying at least two VIRTIS observations targeting the same region close in time with different geometries to be used for triangulation, some efforts have been spent to address this issue, based on DSMC modelling (Fougere et al. 2016b,a). The model was applied to images acquired by VIRTIS-M when the comet was at a heliocentric distance smaller than 1.9 AU, in order to reproduce the observed emissions due to the two species, and their distributions around the nucleus. The 3D model allowed the conversion of the column density, derived from VIRTIS measurements, into production rates of CO_2_ (Q(CO_2_) $\sim 2\cdot 10^{25}~\text{s}^{-1}$) and H_2_O (Q(H_2_O) $\sim 4\cdot 10^{26}~\text{s}^{-1}$) and inferring the more productive areas on the surface from which the emissions originated. As an example, the DSMC result for the most active regions on the 67P surface derived for H_2_O is shown in Fig. 13 (left panel), based on VIRTIS-M observations acquired in April 2015. The model confirmed that the CO_2_ content is higher in the southern hemisphere of comet 67P, with possible sources located in the Imhotep and Khonsu regions. In addition, the evolution of the outgassing as the comet approached the Sun, indicates that water outgassing increased, while a relatively low variation in CO_2_ outgassing was observed, as indicated by the CO_2_ to H_2_O ratio, which decreases moving closer towards the Sun (Fougere et al. 2016b). Close to perihelion (July 8th, 2015 to September 27th, 2015), the emission location of the major species, H_2_O, CO_2_, CO and O_2_ were more correlated with respect to pre-perihelion observations, as derived from DSMC modelling (Fougere et al. 2016a) applied to observations with the high spectral resolution channel of VIRTIS without imaging capabilities, VIRTIS-H.

Simultaneous observations of the fundamental $\nu _{3}$ band of H_2_O with VIRTIS and the H-Lyman $\beta $ band with Alice (Stern et al. 2007) on board Rosetta showed a good correlation of Lyman $\beta $ brightness and water column densities (Chaufray et al. 2017) and were also used to determine an unexpectedly high intensity of atomic line emissions compared to model predictions based on water column density scattering dissociation. This extra-signal was explained with electron dissociative excitation of water molecules located within few km from the nucleus that contributes to the final atomic emissions (Feldman et al. 2015).

The surface temperatures, derived by inverting the long-wavelength part of VIRTIS-M spectra, presented a latitudinal dependence, being higher than expected in the regions in and close to the neck, like Ash, Babi, Hapi, Seth and Ma’at (Tosi et al. 2019). In contrast, at equatorial and southern latitudes (Aker, Anubis, Atum, Imhotep and Kephry), the retrieved surface temperatures were consistent with a black body behaviour. High temperatures might be explained with small-scale roughness of the surface and indirect radiation contributions in topographically concave regions. By combining VIRTIS and MIRO observations, it clearly appears that the active physical processes occur in the uppermost layers of the nucleus. These include also erosion of the first few cm of the surface layer due to the removal of volatiles and dust caused by solar heating. Further work is required to investigate thermal seasonal effects on regions of the 67P surface and link the retrieved surface temperatures to the dust and gas properties in the coma.

During the inbound phase of 67P the spatial correlation between dust and gas shows that the dust emission in the coma was strongly correlated with the presence of H_2_O emissions and was quite different from the distribution of CO_2_ emissions. This indicates that water is the main driver of dust activity in this time period (Rinaldi et al. 2016). This is also generally consistent with observations from the ground, showing that long-term variations in total water production rate correlate with the total dust brightness (Hansen et al. 2016). Despite water is the main driver of dust activity at this period, no strong temporal correlation between total dust brightness and water production rates is found. As discussed in (Tubiana et al. 2019), when 67P is approaching perihelion, the dust activity cannot be understood based on water-driven activity alone. This is likely to be attributed to regional changes of surface properties, volatile content or access to this. This is in agreement with other modelling results on the seasonal evolution of the near-nucleus coma, which show that the correlation observed earlier in the mission, between the observed dust coma and a modelled water coma from a homogeneously sublimating nucleus (e.g. Shi et al. 2018), is significantly degraded.

During the period of the inbound equinox the inner coma radiance was dominated by particles larger than 10 μm with a power law index larger than −3.1. This is consistent with the result found by GIADA (Fulle et al. 2016). Furthermore, the dust did not change its spectral properties and size distribution from March to April 2015 (Rinaldi et al. 2016, 2017). The colour of the dust in the coma has a fairly steep red slope in the visible of about 9 to $12\pm1\%/100~\text{nm}$ from 0.45 to 0.75 μm both on the sunlit side and on the dark side. The reflectance then transitions into a much shallower slope in the infrared from 1 to 2.5 μm with values of $1.7 \pm 0.2 \%/100~\text{nm}$ on the sunlit side and $3 \pm 1\% 100~\text{nm}$ on the dark side. A possible explanation for these values is the presence of dark particles in the coma. No spectral features attributable to the dust could be detected. The colours of the dust do not show any spatial variation, nor any variation with distance from the nucleus. This indicates that the dust ejected from the nucleus into the coma is quite uniform, although it could also be that any changes in its composition or particle size distribution do not affect its colour.

During perihelion 67P’s activity led to rejuvenation of its surface, exposing underlying and mostly primordial material. A comprehensive study of the diffuse coma and outbursts observed with VIRTIS was presented by Bockelée-Morvan et al. (2017, 2019) and Rinaldi et al. (2018). The main results are the particle size distribution in the quiescent coma and the detection of high-temperature grains during outburst. Bockelée-Morvan et al. (2017) modelled the infrared dust emission to explain the quiescent dust coma characterised by a bolometric albedo of 0.13, a colour of 2.5%/100 nm in the IR, and a colour temperature which is 20% in excess with respect to the equilibrium temperature. The best fit for the quiescent coma was achieved with a differential size distribution with a size index of −2.5 to −3, the same values as obtained during the pre-perihelion period (Rinaldi et al. 2017). The observed colour temperature can be attributed either to the presence of sub-micrometre particles made of absorbing material or, alternatively, to fractal agglomerates with sub-micrometre units. The scattering and thermal properties of 67P’s diffuse coma are the same as found for other moderately active comets.

Many outbursts have been observed (e.g. Vincent et al. 2016a; Rinaldi et al. 2018), characterised by a sudden increase of the dust emission, peaking a few minutes later, followed by a smooth decrease of the emission until it returns to the pre-outburst value. The dust material detected during the increase phase of the dust emission is associated with a large increase of the colour temperature (from 300 to 630 K) and a change of the dust colour in the visible and infrared spectra from red to blue, revealing the presence of very small grains ($\leq100~\mbox{nm}$) with a velocity of 20–40 m/s. In addition, the measured large bolometric albedos (∼0.7) indicate bright grains in the ejecta, which could either be silicatic grains, implying thermal degradation of the carbonaceous material, or icy grains (Bockelée-Morvan et al. 2017; Rinaldi et al. 2018). The far-ultraviolet spectrograph, Alice, onboard Rosetta observed the same outburst in the range between 70 and 205 nm. The spectra show dust in the plume that is brighter than the sunlit nucleus, and which displays a strong absorption feature around 170 nm. This feature is characteristic of water ice (Steffl et al. 2016), suggesting that the observed material is likely to consist primarily of icy grains. The absence of a corresponding feature at 3 μm would be consistent with the presence of very small icy dust particles with radii less than 100 nm (Bockelée-Morvan et al. 2017).

## Summary and Outlook

A comet is a highly dynamic object, undergoing a permanent state of change. These changes have to be carefully classified and considered according to their intrinsic temporal and spatial scales. For instance, one can consider the evolution of micro-physical properties on long time scales (years), or large morphological changes associated with outbursts or similar transient events on the scales of minutes. However, the challenge is still to fully understand the processes we usually associate with “typical” or “normal cometary” activity. On the surface and near sub-surface the changing conditions are clearly demonstrated in optical measurements of light scattered by dust lifted from the surface. The gas, considered to be the driving force liberating dust particles, can be observed remotely in a number of wavelengths. For this purpose the latest comet mission Rosetta had “eyes” in the UV (Alice, VIRTIS-M), VIS (OSIRIS, VIRTIS), IR (VIRTIS, OSIRIS), sub-mm (MIRO), and also featured in-situ instruments for the gas composition (ROSINA) and dust properties (GIADA, COSIMA, MIDAS). This allowed the long-term investigation of the inner coma as never observed before from ground or with other space missions. This enabled the identification of different regimes occurring in the inner coma, compared to the outer. One key type of measurement that could not be performed by Rosetta was the determination of the surface temperature with a high-resolution infrared mapping spectrometer sensitive in the wavelength range of the peak of the thermal emission in the 1–3 AU heliocentric range (10–30 micron).

One of the key questions is the characterisation and understanding on the gas and dust sources on the nucleus. This includes their spatial distribution, whether they posses an intrinsic “size”, and whether there is a time scale over which they are activated and/or become dormant. Finally, we want to know the conditions and mechanism of how the dust is actually released from the surface. The final part of a life cycle of a comet, other than e.g. the dynamical ejection from the solar system, is the integral effect of the evolution of such sources.

We have outlined the processes of dust and gas release from the surface and the dynamical aspects of the expansion of the subsequent flows. Driven by the recent Rosetta mission the development of ever more complex and physically precise dynamical models has greatly enriched our understanding of cometary gas and dust dynamics in the innermost coma. The interplay of topography/roughness and global nucleus shape effects, surface compositional inhomogeneities as well as ever-changing illumination and shadowing conditions can no longer be denied. Yet challenges remain. How can we describe the cometary surface such that it can be translated into an adequate ‘effective boundary’ used in dynamical dusty-gas simulations? Or how do we describe physical properties of the dust considering the diversity of particles observed and the fact that each particle is unique in shape, composition etc.?

Individual remote sensing observations are inherently convolved in space and time. Therefore, solving the inverse problem for the characterisation of local sources is highly challenging. In-situ measurements are inherently local, and inverting for local surface sources is a rather ill-posed problem as shown in several publications. The identification of further active areas on the surface is thus of importance. A non-identification might be a consequence of the limited instrument resolution and natural resolution limits within models. Furthermore, the determination of inactive areas or regions has yet to be successful. Are there indeed no inactive areas on a comet? On what scale does activity occur and on what spatial scales are refractories and ice mixed? It is likely that only a new mission can answer these questions.

There does not seem to be a single uniform process behind cometary activity. Rather, activity seems to be the consequence of a variety of erosion processes, including the sublimation of both water ice and more volatile material, but possibly also more exotic processes such as fracture and cliff erosion under thermal and mechanical stress, sub-surface heat storage, and a complex interplay of these processes.

While Rosetta has provided a a wealth of data for volatile species, observations at larger heliocentric distances would help to investigate some of the super-volatile species (like CO_2_ and CO) and their interactions with dust. This would also contribute to our understanding of more distant active objects in our solar system such as some of the Centaurs. For this objective again, a new mission would be required.

The future outlook on how to proceed with the existing Rosetta dataset can be simply put as “synergy”. What is needed is to combine forward models of the different measurements. This includes explicitly a retrospective assimilation methodology borrowed e.g. from Earth meteorology models. Of course, a step in between might be still trying to combine them implicitly, where measurements of other instruments (or results of inversions thereof) can be put as constraints for one another. The 3D and/or 4D modelling of such constrained modelling is still ahead of us, in part due to its high computational demands.

Another key result of Rosetta has been the importance of seasonal effects. The interplay of redeposition and mass transport at the surface, and cometary activity remains elusive. Mass transport from one region to another provides a feedback effect on the activity observed. Icy spots were identified on the surface and investigated for their seasonal variability. They are in agreement with identified active areas on the surface that are supposed to be the origin of the observed emissions in the coma. However, a direct link is still missing. Furthermore, the degree to which observed inhomogeneities of the surface are the result of evolutionary processes versus reflective of the nucleus composition remains to be explored. Thus a detailed monitoring of some regions to investigate their activity and seasonal variability would hold important information. With Rosetta global coverage was privileged instead of a survey of selected interesting regions. Hence, this task is only partly achievable with the current data sets, and a new mission would be required.

The importance of seasons and the nucleus shape for the distribution and temporal evolution of activity implies that the heliocentric evolution of activity can be highly individual for every comet, and generalisations can be misleading. Finally, the large amount of back-fall material suggests that there is no simple relationship between a global gas production rate measured from ground and the “largest liftable grain size” which typically determines the total mass of the ejected dust. This leaves open a big question of how ground based observations can inform our understanding of cometary nuclei.
